# A new application of click chemistry *in situ*: development of fluorescent probe for specific G-quadruplex topology

**DOI:** 10.1038/srep17202

**Published:** 2015-11-25

**Authors:** Ming-Hao Hu, Xiao Chen, Shuo-Bin Chen, Tian-Miao Ou, Meicun Yao, Lian-Quan Gu, Zhi-Shu Huang, Jia-Heng Tan

**Affiliations:** 1 School of Pharmaceutical Sciences, Sun Yat-sen University, Guangzhou, 510006, China

## Abstract

Target-guided synthesis is an approach to drug discovery that allows the target to self-assemble its own binding agents. So far, target-guided synthesis and especially *in situ* click chemistry have attracted extensive attention and have led to the identification of highly potent inhibitors for proteins. In this study, we expand the application of *in situ* click chemistry and present a procedure using this approach to identify selective fluorescent probes for a specific topology of G-quadruplex nucleic acids, the parallel G-quadruplexes. On this basis, compound 15 assembled by triarylimidazole scaffold and carboxyl side chain was a positive hit, demonstrating highly potential in the sensitive and selective detection of parallel G-quadruplexes. Such selective fluorescence response can be rationalized in terms of different binding affinities between 15 and G-quadruplexes. Our work accordingly represents a new development towards the application of *in situ* click chemistry to develop selective fluorescent probes and may also shed light on the search for probes for a specific G-quadruplex topology.

Target-guided synthesis (TGS) is a subset of combinatorial chemistry in which the biological target (protein or nucleic acid) is directly involved in the choice of ligands assembled from a pool of reactive building blocks^1^. TGS can be divided into two major classes: dynamic combinatorial chemistry and kinetic TGS^2^,^3^. In kinetic TGS, the reaction that joins the building blocks is irreversible, and selectivity for some products over others is a function of differential acceleration of that reaction by the target^4^. One approach of kinetic TGS, *in situ* click chemistry, employs the completely biorthogonal [1,3]-dipolar cycloaddition reaction between azides and alkynes and has received much attention^5^,^6^. This azide-alkyne cycloaddition (AAC) reaction is rather slow in a biologically relevant environment but is accelerated when the terminal azide and alkyne groups are held together in close proximity by a given biological target. Because a large kinetic barrier must be overcome in the AAC reaction, the positive hits from *in situ* click chemistry could represent compounds with substantial improvements in binding ability. In past decades, *in situ* click chemistry has become an effective approach to discover new drug candidates, thereby leading to the discovery of numbers of agents that bind nucleic acids and proteins with strong affinity^7^,^8^,^9^,^10^,^11^,^12^,^13^.

G-quadruplexes are unique four-stranded nucleic acid structures formed by guanine-rich sequences in many crucial genomic regions that can be divided into three main topologies: parallel, antiparallel, and hybrid-type structures^14^,^15^. During the past two decades, G-quadruplexes have attracted extensive attention because of their biological significance and potential applications in supramolecular chemistry^16^,^17^, thereby promoting the development of fluorescent probes for the selective detection of these structures^18^,^19^,^20^. Among them, the development of fluorescent probes for a specific G-quadruplex topology is more attractive and challenging than probes only considering the selectivity for G-quadruplexes against single- and double-stranded nucleic acids. Until now, several probes that possess the ability to distinguish different G-quadruplex topologies were reported^21^,^22^,^23^,^24^,^25^,^26^,^27^. However, the rational discovery of such fluorescent probes is rarely reported. Interestingly, in 2012, the *in situ* click chemistry approach was proven to be suitable to enhance the binding affinity of small molecules with a given G-quadruplex^12^. Notably, fluorescence emission of molecules upon binding to G-quadruplexes and their binding affinities were always positively correlated^24^,^28^. Taken together, these results indicate that the *in situ* click chemistry approach might be feasible for the development of fluorescent probes for a specific G-quadruplex topology.

Recently, we have reported a series of triarylimidazole fluorescent probes for selectively detecting G-quadruplexes^24^,^28^. Among them, **IZCM-1** exhibited considerable selectivity for parallel G-quadruplexes, but subsequently, some shortcomings were observed in our studies. First, discrimination between some sequences was not selective enough. For example, the fluorescence quantum yield value of **IZCM-1** for the parallel G-quadruplex c-kit2 was only approximately 3.5-fold higher than that for the antiparallel G-quadruplex HRAS. Meanwhile, the detection limits for some parallel G-quadruplexes were not satisfactory enough. To improve the sensitivity of our probe, we further developed another triarylimidazole probe, **IZCM-7,** for the highly sensitive detection of G-quadruplexes. Nevertheless, discrepancy of fluorescence emission of **IZCM-7** between parallel and non-parallel G-quadruplexes was further reduced. Therefore, the modification strategy of triarylimidazole fluorescent probes must be reconsidered, and we began to examine the possibility of developing more selective fluorescent probes for parallel G-quadruplexes using an *in situ* click chemistry approach. We present a procedure using *in situ* click chemistry to identify selective fluorescent probes for parallel G-quadruplexes. To the best of our knowledge, this is the first example of the development of selective probes for a given nucleic acid topology using an *in situ* click chemistry approach.

## Results and Discussion

### Application of *in situ* click chemistry to identify fluorescent probe for specific G-quadruplex topology

**Figure 1:**
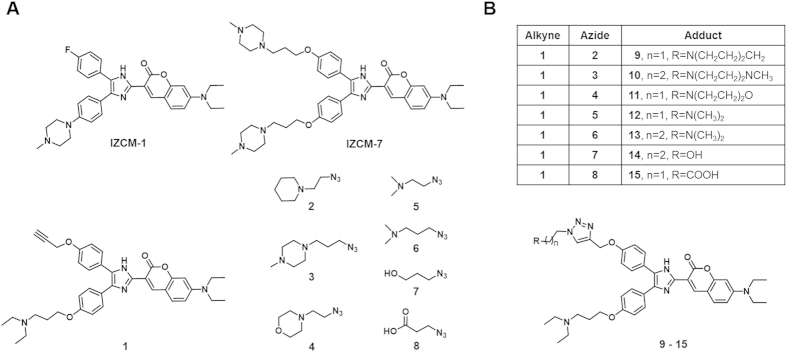
Structures of all the compounds in this article. (**A**) Structures of **IZCM-1**, **IZCM-7**, alkyne **1** and azide building blocks **2**–**8**. (**B**) Adducts **9**–**15** generated by treating alkyne **1** with azides **2**–**8**.

Based on our previous results, c-kit2 G-quadruplex DNA representing a parallel structure and HRAS G-quadruplex DNA representing a non-parallel structure were chosen as templates. A G-quadruplex binding substrate **1** containing an alkyne substituent was used as the anchor molecule. Alkyne **1** was derived from **IZCM-7**, but it contained only one cationic side chain attached to the chromophore, thereby leaving room to modulate the binding properties of the substrate through a click reaction with a series of azides. The *K*_D_ values of **1** to c-kit2 and HRAS were determined by SPR assays as 13.9 μM and 37.9 μM, respectively (see the Supplementary Information), demonstrating that **1** could actually bind to the G-quadruplexes and act as a starting anchor molecule. Azides **2** to **8**, which contain neutral, positively and negatively charged groups that cover electrostatic and hydrogen bonding interaction modes, were employed as complementary reagents for *in situ* click chemistry screening (Fig. 1).

**Figure 2:**
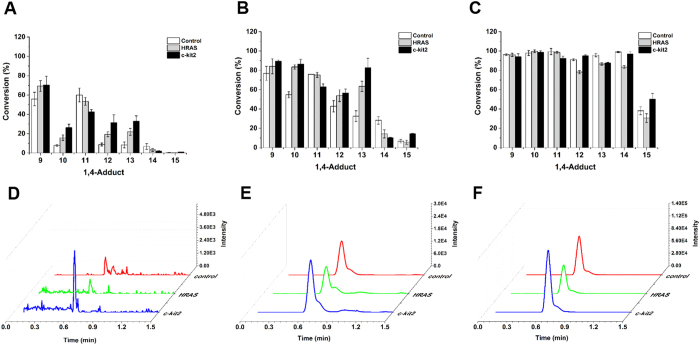
Outputs of *in situ* click chemistry. Upper: conversion of all 1,4-adducts obtained from each pairwise reaction with and without G-quadruplexes at (**A**) 1 h, (**B**) 5 h and (**C**) 24 h. Lower: chromatogram tuned on the mass channel of adduct **15** obtained from the reaction performed with and without G-quadruplexes at (**D**) 1 h, (**E**) 5 h and (**F**) 24 h.

The *in situ* click chemistry experiments were conducted using 96-well microtiter plates. Each well contained a mixture of a G-quadruplex (c-kit2 or HRAS, 12.5 μM), alkyne **1** (12.5 μM), and a given azide reagent (50.0 μM) in Tris-HCl buffer (100 mM KCl, pH = 7.2). In parallel, control experiments without G-quadruplex were performed to test for product formation as a result of background reactivity. We aimed to find a compound that is an *in situ* hit when using parallel c-kit2 as a template but not a hit when using antiparallel HRAS. Next, the microtiter plate was stirred at room temperature for 72 h. Subsequently, the formation of the product was monitored by UPLC using mass spectrometric detection in the positive selected ion mode (UPLC/MS). Nevertheless, a very small amount of the corresponding product in each well was detected, indicating that the AAC reaction was extremely slow even in the presence of G-quadruplex templates. According to the previous research, a Cu^I^ catalyst could accelerate such an ACC reaction and compensate for poor kinetics while preserving high selectivity^12^. Based on this finding, we further modified our *in situ* click chemistry experiments by adding Cu^I^ catalyst to the reactions (Cu^I^ used in our experiments had a slight effect on the G-quadruplex topologies, see the Supplementary Information). Afterwards, we analyzed the reaction mixtures for the generated triazoles by UPLC/MS at three different time points: 1 h, 5 h and 24 h. The results are shown in Fig. 2 (upper). The ACC reactions were greatly accelerated by Cu^I^ catalyst. Compared with the control experiments (with Cu^I^ catalyst but without G-quadruplex templates), G-quadruplex template c-kit2 actually increased the formation of most 1,4-adducts (**9**, **10**, **12**, **13** and **15**) to various extents at 1 h and 5 h, while HRAS promoted the generation of compounds **9**, **10**, **12** and **13**. We also noticed that product formation of all azides with the exception of azide **8** reached almost 100% at 24 h, thereby making it difficult to evaluate the functions of DNA templates. Besides, the conversion rate of **15** was much lower than other 1,4-adducts. Such lower conversion could be ascribed to the weaker reactivity of 3-azidopropanoic acid because we had the same findings in our further synthesis of all the individual adduct (prepared on a preparative scale using the CuI process, see the Supplementary Information). In the present *in situ* click chemistry experiments, control experiments without G-quadruplex were performed. Thus, various reactivity of different azides would not affect the judgment on the hits. Taking our findings together, among all the 1,4-adducts, compound **15**, which resulted from the cycloaddition of **1** and **8**, was distinctive because c-kit2 accelerated the reaction, but HRAS hindered product formation at each of the three time points (Fig. 2, lower), suggesting that **15** prefers to bind c-kit2 with a much lower *K*_D_ than HRAS and could possibly act as a favorable fluorescent probe for parallel G-quadruplexes.

To provide a rationale regarding the template effect of G-quadruplexes, we analyzed the binding affinities of starting alkyne **1** and each individual adduct (prepared on a preparative scale using the Cu^I^ process, see the Supplementary Information) to c-kit2 and HRAS by SPR experiments. As shown in Table 1, without exception, the *in situ* hit compounds displayed higher binding affinities for G-quadruplexes than alkyne **1**. Compound **15**, which was a hit in the presence of c-kit2 but not HRAS, exhibited much stronger binding affinity to c-kit2 than to HRAS (see the Supplementary Information). The greatest difference in binding affinities between c-kit2 and HRAS distinguished **15** from other adducts, causing it to be a favorable fluorescent probe for parallel G-quadruplexes. Some other adducts such as **9**, **10**, **12**, and **13**, which were all *in situ* hits in the presence of HRAS and c-kit2, did not display remarkable differences in the binding affinities for c-kit2 and HRAS. However, the remaining two adducts, **11** and **14**, which were not hits from the *in situ* click chemistry approach, displayed slight enhancements in the binding affinities to c-kit2 and HRAS. These results demonstrate that compounds formed *in situ* were very likely to be strong G-quadruplex-interacting molecules, whereas the reverse was not true, leading to “false negatives”^9^. Of the eight azides, positively charged (amines) azide-derived triazoles showed the weakest discrimination between c-kit2 and HRAS, the negatively charged (carboxylic acid) azide-derived products displayed excellent discrimination between the two G-quadruplexes. The neutral triazoles (hydroxyl) possessed a medium capability to discriminate between G-quadruplexes. In general, this trend agreed with results observed in the copper-catalyzed *in situ* click chemistry approach.

Simultaneously, the fluorescence quantum yields of all 1,4-adducts and alkyne **1** in the presence of c-kit2 and HRAS were detected (Table 2). As expected, the general trend of fluorescence enhancements is compatible with the affinities of the 1,4-adducts binding to G-quadruplexes. Among all the 1,4-adducts, compound **15** presented the highest selectivity between c-kit2 and HRAS. Accordingly, these results reinforced our design idea to discover a fluorescent probe specific for parallel G-quadruplexes using an *in situ* click chemistry approach.

### Fluorescence studies of compound 15 interactions with nucleic acids

**Figure 3:**
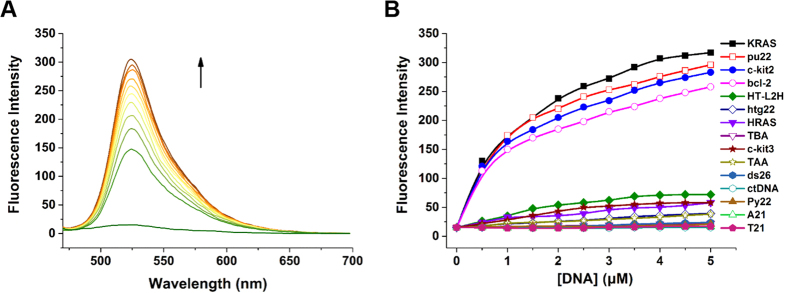
Fluorescence properties of 15 with different nucleic acids. (**A**) The fluorescence titration of 0.5 μM compound **15** with the stepwise addition of c-kit2 (arrow: 0–5 μM) in 10 mM Tris-HCl buffer, 100 mM KCl, pH 7.2. (**B**) The fluorescence intensity enhancement of 0.5 μM **15** at 525 nm against the sample concentrations, λ_ex_ = 450 nm.

To further determine the selectivity of compound **15** for parallel G-quadruplexes, its fluorescence properties with various G-quadruplexes and other nucleic acids were explored using a fluorescence titration assay. As shown in Fig. 3A, **15** alone in buffer displayed extremely weak emission. Upon gradual addition of c-kit2, an emission peak at approximately 525 nm appeared and was significantly enhanced. This significant increase in fluorescence was also observed when **15** was treated with the G-quadruplexes KRAS, pu22 and bcl-2, which had all been determined to form parallel structures (Fig. 3B). In contrast, we observed much weaker fluorescence enhancement for the hybrid-type G-quadruplex structures HT-L2H and htg22 and for antiparallel G-quadruplexes including HRAS, TBA and c-kit3 under experimental conditions. In addition, negligible fluorescence enhancement was observed when **15** was titrated with single-stranded DNA (Py22, A21 and T21), double-stranded DNA (ds26 and ctDNA), and triplex DNA (TAA).

The fluorescence quantum yield values of **15** for different nucleic acids are summarized (see the Supplementary Information). Such data were in agreement with the results of a titration experiment showing that fluorescence enhancement was always more pronounced for parallel G-quadruplexes. The quantum yield of **15** for c-kit2 reached 0.44. This value is approximately 7.5 times higher than that for HRAS. Moreover, such discrepancy was more significant than that of **IZCM-1**, showing that the quantum yield for c-kit2 was only 3.5-fold higher than that for HRAS^24^. On average, the quantum yield of **15** for parallel G-quadruplexes was 10.4 times higher than that for other types of G-quadruplexes. In contrast, the values were 8.0 and 1.3 for **IZCM-1** and **IZCM-7**, respectively^24^,^28^.

**Figure 4:**
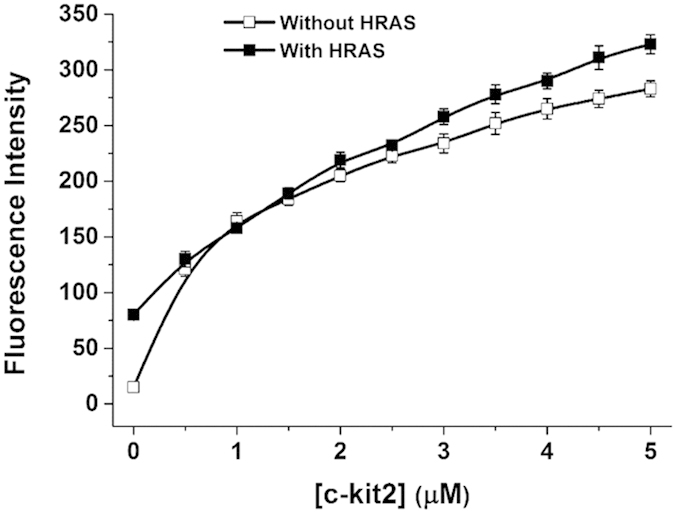
Competitive fluorescence titrations. Fluorescence titrations of 0.5 μM **15** with the stepwise addition of c-kit2 with and without 5 μM HRAS in 10 mM Tris-HCl buffer, 100 mM KCl, pH 7.2.

Furthermore, competition titrations were performed to confirm the selective fluorescence response of **15** binding to parallel G-quadruplexes. As shown in Fig. 4, when gradually adding c-kit2 into the solution containing **15** and 5 μM HRAS, the enhanced fluorescence trend was similar to that observed in the experiment without HRAS. These results suggested that **15** has promising utility in the selective detection of parallel G-quadruplexes.

Moreover, we evaluated the detection limits of **15** for parallel G-quadruplexes. The LOD values of **15** for parallel G-quadruplexes (KRAS, pu22 and c-kit2) in solution were approximately 10 nM, which were better than those of **IZCM-1** and similar to those of **IZCM-7** (see the Supplementary Information), indicating **15** was a more favourable sensitive fluorescent probe compared with lead **IZCM-1**.

As an example of application, we set to demonstrate the potential of compound **15** as a topology-specific staining reagent for parallel G-quadruplexes in electrophoresis gels. We employed KRAS, pu22, c-kit2, htg22, HRAS, c-kit3 and ds26 in the experiments. After electrophoresis, the polyacrylamide gels were immersed in 4 μg/mL compound **15** staining solution for 20 minutes and commercial SYBR^®^ Green I was used as a benchmark. We were only able to detect bands corresponding to parallel G-quadruplexes KRAS, pu22 and c-kit2, whereas we found no staining for hybrid-type G-quadruplex htg22, antiparallel G-quadruplex HRAS and c-kit3, and double-stranded DNA ds26. In contrast, SYBR^®^ Green I stained all the nucleic acid bands in the same gel (see the Supplementary Information). These results further highlighted the feasibility of using **15** as a selective fluorescent stain for parallel G-quadruplexes.

### Binding model of compound 15 interactions with G-quadruplexes

**Figure 5:**
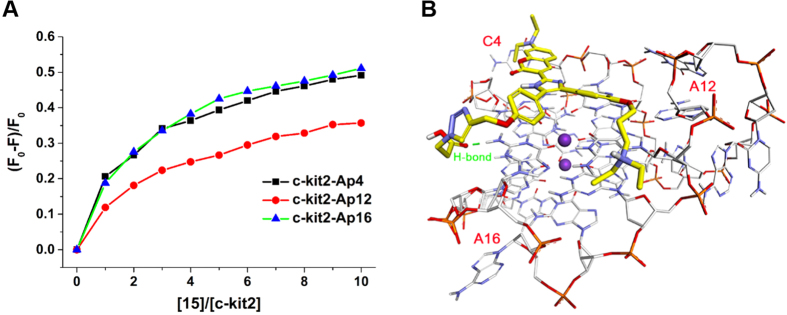
Studies of binding model of 15 to parallel G-quadruplex c-kit2. (**A**) Plot of normalized fluorescence intensity at 375 nm of 1 μM 2-Ap individually labeled c-kit2 versus binding ratio of [**15**]/c-kit2], λ_ex_ = 305 nm. (**B**) Binding model of compound **15** to parallel G-quadruplex c-kit2 (PDB ID: 2KQR).

Modification of 2-aminopurine (2-Ap) in different loops has been widely used to estimate the binding mode of small molecules with G-quadruplexes^24^,^29^. To gain more details on the interactions of **15** with parallel G-quadruplexes, we performed fluorescence experiments using the parallel G-quadruplex c-kit2 with 2-Ap substitutions at position 4, 12, and 16. The antiparallel G-quadruplex HRAS with 2-Ap substitutions at position 7, 16, and 21 was used as well. It was found that the fluorescence intensities of Ap4, Ap12 and Ap16 in c-kit2 were significantly affected upon the addition of **15** (Fig. 5A), indicating that **15** had close contacts with these bases and probably stretched over the whole G-quartet plane of c-kit2 accordingly. In contrast, the fluorescence intensities of Ap7, Ap16 and Ap21 in HRAS were slightly affected by **15** (see the Supplementary Informations), which proved that **15** might have very weak interaction with HRAS. Such results were consistent with the SPR studies showing **15** exhibited much stronger binding affinity to c-kit2 than to HRAS. Furthermore, we also carried out these 2-Ap experiments using compound **10** that had an *N*-methylpiperazine side chain instead of the carboxyl side chain. As compared to **15**, we observed that the fluorescence of 2-Aps in both c-kit2 (at position 4, 12 and 16) and HRAS (at position 7 and 21) were remarkably affected upon addition of **10** (see the Supplementary Information), indicating that strong interactions occurred between **10** and the two G-quadruplexes, mainly because the positive *N*-methylpiperazine side chain would increase electrostatic interactions with HRAS. Notably, Ap7 and Ap21 in HRAS were positioned in two lateral loop region close to the same G-quartet. The distinct responses of 2-Aps in HRAS with the addition of **10** and **15** indicated that its lateral loop would hinder **15** from stacking onto the corresponding G-quartet, probably due to electrostatic repulsion between the negatively charged carboxyl side chain on **15** and phosphate groups in loop region, and thus leading to their loose interaction and the subsequent weak fluorescence response of **15** in the presence of antiparallel G-quadruplex HRAS.

Based on the findings of 2-Ap experiments, molecular docking studies were performed to illustrate the binding of **15** to parallel G-quadruplex c-kit2. The parallel NMR G-quadruplex structure for c-kit2 was used as the template^30^. Molecular model of **15** with c-kit2 was generated by docking study. As shown in Fig. 5B, compound **15** perfectly stacked on the terminal G-quartet plane of c-kit2 by a π-π interaction. The three outstretched side arms on the triarylimidazole scaffold bound to the grooves of G-quadruplex c-kit2. Notably, the carboxyl side chain stretched into the groove of c-kit2 and then interacted with the guanine base via hydrogen bond, leading to the formation of a tight complex of **15** and c-kit2. Considering the binding of **15** to antiparallel G-quadruplex HRAS, these interactions could not occur because the negatively charged carboxyl side chain would hinder **15** from stacking onto the G-quartet. Collectively, these findings offered us understandable explanation about the selectivity of **15** to parallel G-quadruplexes.

## Conclusions

In summary, we have successfully employed an *in situ* click chemistry approach to develop a more sensitive fluorescent probe for parallel G-quadruplexes based on the triarylimidazole scaffold. Based on our results, **15** was chosen as the most promising fluorescent probe, demonstrating excellent application in the selective and sensitive detection of parallel G-quadruplexes compared with the lead compounds **IZCM-1** and **IZCM-7**. Such selective fluorescence response can be rationalized in terms of different binding affinities between **15** and G-quadruplexes. Taken together, our findings represent a new development towards the application of *in situ* click chemistry to develop selective fluorescent probes and may also shed light on the search for probes for a specific G-quadruplex topology. Furthermore, this study represents an important first step that can be used in the discovery of selective probes that target a given G-quadruplex structure with an independent sequence. It should also be noted that G-quadruplexes usually interact with proteins *in vivo*, accordingly their 3D structures will be different from those *in vitro*. It is more worthy to find probes targeting these *in vivo* structures. Undoubtedly, a lot of work should be done to ensure the feasibility of the *in situ* click chemistry approach in various situations. Further investigations are now underway.

## Methods

### Compound synthesis

All chemicals were purchased from commercial sources unless otherwise specified. Detailed description of the synthesis of each compound can be found in the Supplementary Information. Their structure and purity were confirmed by ^1^H and ^13^C NMR spectrometry, HRMS spectrometry, and HPLC analysis.

### Materials

All oligonucleotides used in this study (see the Supplementary Information) were purchased from Invitrogen (China) and Sangon (China). Calf thymus DNA (ctDNA) was purchased from Sigma-Aldrich (Singapore). All the oligonucleotides and ctDNA were dissolved in relevant buffer. Their concentrations were determined from the absorbance at 260 nm, respectively on the basis of respective molar extinction coefficients using NanoDrop 1000 Spectrophotometer (Thermo Scientific, USA). To obtain G-quadruplex formation, oligonucleotides were annealed in relevant buffer containing KCl by heating to 95 °C for 5 min, followed by gradual cooling to room temperature. The oligonucleotides were engaged in G-quadruplex formation, as determined by circular dichroism (CD) measurements. Stock solutions of compounds (10 mM) were dissolved in DMSO and stored at −80 °C. Further dilutions of samples to working concentrations were made with relevant buffer immediately prior to use.

### CD Studies

CD studies were performed on a Chirascan circular dichroism spectrophotometer (Applied Photophysics, UK). A quartz cuvette with a 4 mm path length was used for the recording of spectra over a wavelength range of 230–330 nm with a 1 nm bandwidth, 1 nm step size and time of 0.5 s per point. The DNA samples were set at the concentration of 10 μM.

### Cu^I^-catalyzed *in situ* click chemistry

5 μL of G-quadruplex DNA solution at 100 μM (c-kit2 or HRAS), or 10 mM Tris-HCl buffer (PH 7.2, 100 mM KCl), was added to a 96-well microtiter plate. Then, 5 μL of **1** (100 μM) and 1 μL of azide **2**–**8** (1 mM) were added to the mixture (each azide had a different well in the microtiter plate), followed by the addition of 0.5 μl of CuSO_4_·5H_2_O (10 mM) and 0.5 μl of aq. sodium ascorbate (10 mM). 8 μL of Tris-HCl buffer was added to fix the final solution at 20 μL. The microtiter plate was stirred at room temperature for hours before being quenched by adding 180 μL of methanol. The mixtures were then transferred to 1.5 mL Eppendorf tubes and centrifuged at 16000 rpm for 10 min at 4 °C. 10 μL of each supernatant was injected into the UPLC-MS/MS system.

### UPLC-MS/MS method

The chromatography was performed on an Acquity UPLC system (Waters Corp., Milford, MA, USA) with a binary solvent manager, an autosampler (set at 4 °C) and a column oven (set at 25 °C). A BEH C18 column (2.1 × 50 mm i.d., 1.7 μm; Waters, Wexford, Ireland) preceded by a BEH C18 VanGuard ™ pre-column (2.1 × 5 mm i.d., 1.7 μm, Waters, Wexford, Ireland) was employed for the sample separation. The mobile phase composed of 90% methanol and 10% water was used in isocratic mode at a flow rate of 0.35 mL/min. The run-time was 1.5 min. The injection volume was 10 μL.

For mass spectrometry, detection and quantification of analytes were carried out on a triple quadrupole tandem mass spectrometer (Waters Quattro Premier XE, Micromass MS Technologies, Manchester, UK). The electrospray ionization (ESI) source was set in positive ionization mode. Multiple reaction monitoring mode was performed. The optimal MS parameters were as follows: capillary voltage, 3.0 kV; cone voltage, 40 V; source temperature, 120 °C. Ultrahigh-purity nitrogen and argon were used as desolvation gas (700 L/h) and collision gas (0.21 mL/min), respectively. Masslynx™ 4.1 software was used to collect and process data. The conversion of each adduct formation was determined with area normalization method.

### SPR Studies

SPR measurements were performed on a ProteOn XPR36 Protein Interaction Array system (Bio-Rad Laboratories, CA) using a Streptavidin-coated GLH sensor chip. Biotinylated oligonucleotides (SPR-HRAS and SPR-c-kit2) were attached to the chip. In a typical experiment, biotinylated DNA was folded in filtered and degassed running buffer (50 mM Tris-HCl, 100 mM KCl, pH 7.2). The DNA samples were then captured (about 1000 RU) in five flow cell, leaving one flow cell as a blank. Solutions of the compounds were prepared with running buffer through serial dilutions of stock solution. Five concentrations were injected simultaneously at a flow rate of 50 μL/min for 400 s of association phase, followed with 500 s of dissociation phase at 25 °C. The GLH sensor chip was regenerated with short injection of 1 M KCl between consecutive measurements. The final graphs were obtained by subtracting blank sensorgrams from different DNA sensorgrams. Data were analyzed with ProteOn manager software, using the Langmuir model for fitting kinetic data.

### UV-Vis Spectroscopic Studies

UV-Vis spectroscopic studies were performed on a UV-2450 spectrophotometer (Shimadzu, Japan) using 1 cm path length quartz cuvette. All the oligonucleotides were firstly prepared through heating at 95 °C for 5 min followed with slow cooling to room temperature. Small aliquots of a stock solution of oligonucleotide were added into the solution containing compounds at fixed concentration (1 μM) in Tris-HCl buffer (10 mM, pH 7.2) with 100 mM KCl. The final concentration of oligonucleotide was 10 μM. The intensities of absorbance at 450 nm were recorded, preparing for further determination of fluorescence quantum yields.

### Fluorescence Studies

Fluorescence studies were performed on a LS-55 luminescence spectrophotometer (Perkin-Elmer, USA). A quartz cuvette with 2 mm × 10 mm path length was used for the spectra recorded at 5 nm excitation and emission slit widths unless otherwise specified.

For titration experiment, all oligonucleotides were firstly prepared through heating at 95 °C for 5 min followed with slow cooling to room temperature. Small aliquots of a stock solution of sample (oligonucleotides, ctDNA) were added into the solution containing the compounds at fixed concentration (1 μM) in Tris-HCl buffer (10 mM, pH 7.2) with 100 mM KCl. The final concentration of sample was varied from 0 to 5 μM. After each addition of sample, the reaction was stirred and allowed to equilibrate for at least 1 min and fluorescence measurement was taken at Ex 450 nm.

The fluorescence quantum yield (*Φ*_F_) of all the compounds was calculated relative to a standard solution of rhodamine 123 in ethanol (*Φ*_F_ = 0.90) and was determined using the following formula: *Φ*_*u*_ = *Φ*_*s*_ (*A*_*u*_/*A*_*s*_) × (*I*_*u*_/*I*_*s*_), where *Φ* is the fluorescence quantum yield*, I* is the measured integrated emission intensity, and *A* is the optical density (absorbance). The *u* refers to the compound of unknown quantum yield, and *s* refers to the reference compound (Rohdamine 123) of known quantum yield. The fluorescence spectra were recorded at 5 nm excitation and emission slit widths for the determination of *Φ*.

The LOD values of **15**, **IZCM-1** and **IZCM-7** for different nucleic acids in solution were calculated on the basis of the equation LOD = *K* × *S*_*b*_/*m*. The *K* value is generally taken to be 3 according to the IUPAC recommendation. The *S*_*b*_ value represents the standard deviation for multiple measurements (*n* = 20) of blank solution. The *m* value is the slope of the calibration curve, which was derived from the linear range of a fluorescence titration curve with different nucleic acids and standards for the sensitivity of this method.

### Gel Electrophoresis Studies

Different oligonucleotides were loaded onto a 20% bisacrylamide gel in 1 × TBE buffer containing 100 mM KCl and electrophoresed at 4 °C. The oligonucleotides were stained with compound **15** (4 μg/mL, 20 minutes), and then by the commercial staining agent SYBR^®^ Green I (1×, 20 minutes). DNA fragments were visualized under UV light and photographed by using AlphaImager EC (ProteinSimple).

### 2-Ap Titration Experiments

Compound **15** were added into the solution containing 2-Ap-labeled oligonucleotides at fixed concentration (1 μM) in Tris-HCl buffer (10 mM, pH 7.2) with 100 mM KCl. The final concentration of **15** was varied from 0 to 10 μM. After each addition of **15**, the reaction was stirred and allowed to equilibrate for at least 1 min and fluorescence measurement was taken at Ex 305 nm.

### Molecular Docking Process

The structures of compound **15** were constructed and optimized with Gaussian 03 using the HF/6-31G* basis set. The parallel c-kit2-derived NMR G-quadruplex structure was used as the templates (PDB ID: 2KQR) for the docking studies. The docking simulations were performed using Schrodinger software for the binding site based on the reference compounds.

## Additional Information

**How to cite this article**: Hu, M.-H. *et al.* A new application of click chemistry *in situ*: development of fluorescent probe for specific G-quadruplex topology. *Sci. Rep.* **5**, 17202; doi: 10.1038/srep17202 (2015).

## Supplementary Material

Supplementary Information

## Figures and Tables

**Table 1:** Summary of *in situ* click chemistry experiments and dissociation constants.

Compound	*In situ* hit^a^ (HRAS)	*In situ* hit^a^ (c-kit2)	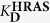 (μM)	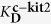 (μM)
**9**	Yes	Yes	6.0	1.8
**10**	Yes	Yes	5.6	2.0
**11**	No	No	8.0	4.8
**12**	Yes	Yes	7.1	2.7
**13**	Yes	Yes	12.9	4.3
**14**	No	No	25.9	3.1
**15**	No	Yes	−b	6.1
**1**	−	−	37.9	13.9

^a^*In situ* hit represents the compound whose formation was accelerated by addition of the G-quadruplex template.

^b^No significant binding was found for addition of up to 40 μM ligand.

**Table 2:** Fluorescence quantum yields of all compounds with G-quadruplexes^*a*^.

Compound^a^	*Φ*_F_ (HRAS)	*Φ*_F_ (c-kit2)	Ratio^b^
**9**	0.420	0.454	1.1
**10**	0.428	0.451	1.1
**11**	0.410	0.443	1.1
**12**	0.400	0.441	1.1
**13**	0.408	0.438	1.1
**14**	0.339	0.413	1.2
**15**	0.059	0.440	7.5
**1**	0.292	0.373	1.3

^a^1 μM of each compound and 10 μM of G-quadruplex were used in the determination of *Φ*_F_.

^b^Ratio means the ratios of *Φ*_F_ (c-kit2) to *Φ*_F_ (HRAS).
