# Supplementary material for: A new application of click chemistry in situ: development of fluorescent probe for specific G-quadruplex topology
Source: Sci Rep. 2015 Nov 25;5:17202. doi: 10.1038/srep17202 (PMC4658637; doi:10.1038/srep17202)
Supplement: Supplementary Information [file srep17202-s1.pdf]

Supplementary Information

A new application of click chemistry in situ: development of fluorescent probe for specific G-quadruplex topology

Ming-Hao Hu, Xiao Chen, Shuo-Bin Chen, Tian-Miao Ou, Meicun Yao,

Lian-Quan Gu, Zhi-Shu Huang & Jia-Heng Tan*

School of Pharmaceutical Sciences, Sun Yat-sen University, Guangzhou 510006, China

1. Experimental Section S2

1.1 Synthesis and Characterization S2

Synthesis of compounds 1-15 for the in situ click chemistry S2-7

Scheme S1. Synthesis of alkyne **1**, azides **2-8** and adducts **9-15** S2

Figure S1-32. ^{1}H NMR, ^{13}C NMR, HRMS spectrum and HPLC analysis of compound **1** and **9-15.** S8-23

2. Other Supporting Table and Graphs S24

Table S1. DNA samples used in the present study S24

Figure S33. SPR sensorgrams of alkyne **1** with G-quadruplex c-kit2 and HRAS S25

Figure S34. CD spectra of G-quadruplex samples with and without Cu^{I} S25

Figure S35. SPR sensorgrams of compound **15** with G-quadruplex c-kit2 and HRAS S25

Table S2. The fluorescence quantum yields of **15** with different nucleic acids S26

Table S3. Detection limits of **15**, **IZCM-1** and **IZCM-7** for different G-quadruplexes in solution S26

Figure S36. Electrophoresis staining of DNA samples by compound **15** and SYBR® Green I S27

Figure S37. Fluorescence titrations of 2-AP labeled HRAS with addition of compound **15** S27

Figure S38. Fluorescence titrations of 2-AP labeled G-quadruplexes with addition of compound **10** S28

1. Experimental Section

1.1 Synthesis and Characterization

Scheme S1. Synthesis of azides **2-8**, alkyne **1**, and adducts **9-15** for the in situ click chemistry.

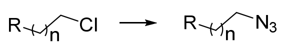

2, n=1, R=N(CH_2CH_2)$_2$$CH_2$
3, n=2, R=N(CH_2CH_2)$_2$$NCH_3$
4, n=1, R=N(CH_2CH_2)$_2$O
5, n=1, R=N(CH_3)$_2$
6, n=2, R=N(CH_3)$_2$
7, n=2, R=OH
8, n=1, R=COOH

Reagents and conditions: NaN_3, acetonitrile or H_2O, 80°C, overnight.

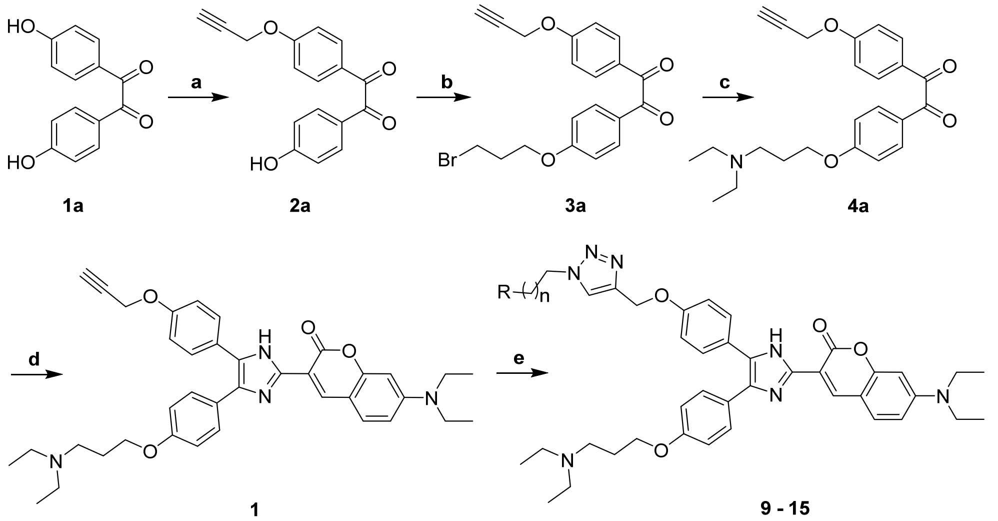

9, n=1, R=N(CH_2CH_2)$_2$$CH_2$
10, n=2, R=N(CH_2CH_2)$_2$$NCH_3$
11, n=1, R=N(CH_2CH_2)$_2$O
12, n=1, R=N(CH_3)$_2$
13, n=2, R=N(CH_3)$_2$
14, n=2, R=OH
15, n=1, R=COOH

Reagents and conditions: (a) Propargyl bromide, K_2CO_3, DMF, 80°C, 5 hours; (b) 1,3-Dibromopropane, K_2CO_3, acetonitrile, reflux, 5 hours; (c) Diethylamine, K_2CO_3, acetonitrile, reflux, 4 hours; (d) 7-Diethylaminocoumarin-3-aldehyde, NH_4OAc, AcOH, reflux, 10 hours. (e) Azide **2-8**, $CuSO_4·5H_2O$, sodium ascorbate, *t*-BuOH/H_2O, 80°C, overnight.

General remarks: ^{1}H and ^{13}C NMR spectra were recorded by using TMS as the internal standard in methanol-d_4 or $CDCl_3$ or DMSO-d_6 at 400 MHz and 101 MHz, respectively, with a Bruker BioSpin GmbH spectrometer. Mass spectra (MS) were recorded on a Shimadzu LCMS-2010A instrument with an ESI or ACPI mass selective detector and high resolution mass spectra (HRMS) were recorded on a Shimadzu LCMS-IT-TOF. Melting points (m.p.) were determined by using an SRS OptiMelt automated melting point instrument without correction. Flash column chromatography was performed with silica gel (200–300 mesh) purchased from Qingdao Haiyang Chemical Co. Ltd. The purity of the synthesized compound was confirmed to be higher than 95% by using analytical HPLC performed with a dual pump Shimadzu LC-20 AB system equipped with a Ultimate XB-C18 column (4.6 × 250 mm, 5 μm) and eluted with methanol-water (80:20) containing 0.1% TFA at a flow rate of 1.0 mL/min. All chemicals were purchased from commercial sources unless otherwise specified. All the solvents were of analytical reagent grade and were used without further purification.

1-(2-chloroethyl)piperidine (2): 1-(2-chloroethyl)piperidine hydrochloride (2.0 g, 10.9 mmol) and NaN_3 (2.1 g, 32.5 mmol) were dissolved in water and allowed to stir at 80 °C overnight. Then, this solution was cooled down and basified by addition of a 2 M NaOH solution, and the mixture was extracted 3 times with Et_2O. Organic layers were collected, dried over Na_2SO_4 and the solvent was evaporated to afford the product as a pale yellow oil (0.81 g, 48% yield). ^{1}H NMR (400 MHz, $CDCl_3$) δ 3.27 (t, J = 6.2 Hz, 2H), 2.48 (t, J = 6.2 Hz, 2H), 2.42 – 2.24 (m, 4H), 1.58 – 1.47 (m, 4H), 1.44 – 1.31 (m, 2H).

1-(3-azidopropyl)-4-methylpiperazine (3): 1-(3-chloropropyl)-4-methylpiperazine hydrochloride (2.3 g, 10.9 mmol) and NaN_3 (2.1 g, 32.5 mmol) were dissolved in acetonitrile/water (1:1) and allowed to stir at 80 °C overnight. Then, this solution was cooled down and basified by addition of a 2 M NaOH solution, and the mixture was extracted 3 times with Et_2O. Organic layers were collected, dried over Na_2SO_4 and the solvent was evaporated to afford the product as a brown oil (0.66 g, 33% yield). ^{1}H NMR (400 MHz, $CDCl_3$) δ 3.34 (t, J = 6.8 Hz, 2H), 2.60 – 2.35 (m, 8H), 2.29 (s, 3H), 2.04 – 1.93 (m, 2H), 1.82 – 1.71 (m, 2H).

4-(2-azidoethyl)morpholine (4): 4-(2-chloroethyl)morpholine hydrochloride (2.0 g, 10.9 mmol) and NaN_3 (2.1 g, 32.5 mmol) were dissolved in acetonitrile/water (1:1) and allowed to stir at 80 °C overnight. Then, this solution was cooled down and basified by addition of a 2 M NaOH solution, and the mixture was extracted 3 times with Et_2O. Organic layers were collected, dried over Na_2SO_4 and the solvent was evaporated to afford the product as a brown oil (0.75 g, 44% yield). ^{1}H NMR (400 MHz, $CDCl_3$) δ 3.75 – 3.62 (m, 4H), 3.33 (t, J = 5.9 Hz, 2H), 2.56 (t, J = 5.9 Hz, 2H), 2.52 – 2.40 (m, 4H).

2-azido-N,N-dimethylethanamine (5): 2-chloro-N,N-dimethylethanamine hydrochloride (1.6 g, 10.9 mmol) and NaN_3 (2.1 g, 32.5 mmol) were dissolved in water and allowed to stir at 80 °C overnight. Then, this solution was cooled down and basified by addition of a 2 M NaOH solution, and the mixture was extracted 3 times with Et_2O. Organic layers were collected, dried over Na_2SO_4 and the solvent was evaporated to afford the product as a pale yellow oil (0.53 g, 43% yield). ^{1}H NMR (400 MHz, $CDCl_3$) δ 3.38 – 3.21 (m, 2H), 2.53 – 2.39 (m, 2H), 2.22 (s, 6H).

3-azido-N,N-dimethylpropan-1-amine (6): 3-chloro-N,N-dimethylpropan-1-amine hydrochloride (1.7 g, 10.9 mmol) and NaN_3 (2.1 g, 32.5 mmol) were dissolved in water and allowed to stir at 80 °C overnight. Then, this solution was cooled down and basified by addition of a 2 M NaOH solution, and the mixture was extracted 3 times with Et_2O. Organic layers were collected, dried over Na_2SO_4 and the solvent was evaporated to afford the product as a pale yellow oil (0.59 g, 42% yield). ^{1}H NMR (400 MHz, $CDCl_3$) δ 3.33 (t, J = 6.8 Hz, 2H), 2.35 (t, J = 7.2 Hz, 2H), 2.23 (s, 6H), 1.80 – 1.69 (m, 2H).

3-azidopropan-1-ol (7): 3-chloropropan-1-ol (2.0 mL, 23.9 mmol) was dissolved in acetonitrile (100 mL). NaN_3 (4.7 g, 71.7 mmol) was added to the solution and the mixture was stirred under reflux overnight. Acetonitrile was then removed under reduced pressure and the resulting residue was suspended in ethyl acetate (50 mL) and extracted with 0.1 N HCl (3 × 50 mL), water (3 × 50 mL) and brine (1 × 50 mL). The organic layer was dried over Na_2SO_4 and concentrated to afford the product as a pale yellow oil (0.87 g, 36% yield). ^{1}H NMR (400 MHz, $CDCl_3$) δ 3.74 (t, J = 5.9 Hz, 2H), 3.61 (t, J = 6.3 Hz, 2H), 2.44 (s, 1H), 2.00 – 1.89 (m, 2H).

3-azidopropanoic acid (8): 3-chloropropanoic acid (2.0 mL, 23.4 mmol) was dissolved in acetonitrile (100 mL). NaN_3 (4.6 g, 70.2 mmol) was added to the solution and the mixture was stirred under reflux overnight. Acetonitrile was then removed under reduced pressure and the resulting residue was suspended in ethyl acetate (50 mL) and extracted with 0.1 N HCl (3 × 50 mL), water (3 × 50 mL) and brine (1 × 50 mL). The organic layer was dried over Na_2SO_4 and then concentrated to afford the product as a pale yellow oil (1.05 g, 39% yield). ^{1}H NMR (400 MHz, $CDCl_3$) δ 3.52 (t, J = 6.4 Hz, 2H), 2.58 (t, J = 6.4 Hz, 2H).

1-(4-hydroxyphenyl)-2-(4-(prop-2-yn-1-yloxy)phenyl)ethane-1,2-dione (2a): Propargyl bromide (0.20 mL, 2.5 mmol) was gradually added to a solution of 4,4'-dihydroxybenzil (1.94 g, 8.0 mmol) and anhydrous K_2CO_3 (0.69 g, 5.0 mmol) in 20 mL dry DMF. The resulting mixture was heated under 80 °C for 5 hours, and then the remaining solution was filtered. After that, 50 mL water was added to the mixture and then was treated with 3.0 M HCl (aq) to reach the pH of 5. The mixture was then extracted by ethyl acetate (3 × 50 mL). The organic layer was dried over Na_2SO_4 and concentrated. Afterwards, the crude product was purified by flash gel chromatography with petroleum ether/ethyl acetate (3:1) as elution solvents to give a pale yellow solid (0.33 g, 48% yield). ^{1}H NMR (400 MHz, DMSO-d_6) δ 10.88 (s, 1H), 7.87 (d, J = 8.6 Hz, 2H), 7.78 (d, J = 8.4 Hz, 2H), 7.19 (d, J = 8.5 Hz, 2H), 6.95 (d, J = 8.4 Hz, 2H), 4.96 (s, 2H), 3.67 (s, 1H). ^{13}C NMR (101 MHz, DMSO-d_6) δ 194.33, 193.77, 164.54, 162.97, 132.86, 132.34, 126.55, 124.49, 116.64, 116.04, 79.51, 78.85, 56.41. ESI-MS m/z: 281.1 $[M+H]^+$.

1-(4-(3-bromopropoxy)phenyl)-2-(4-(prop-2-yn-1-yloxy)phenyl)ethane-1,2-dione (3a): To a stirred suspension of **2a** (0.28 g, 1.0 mmol) and anhydrous K_2CO_3 (0.28 g, 2.0 mmol) in 20 mL dry acetonitrile, 1,3-dibromopropane (0.51 mL, 5.0 mmol) was added. The resulting mixture was heated under reflux for 5 hours, and then the remaining solution was filtered. After concentration, the crude product was purified by flash gel chromatography with petroleum ether/ethyl acetate (4:1) as elution solvents to give a white solid (0.31 g, 77% yield). ^{1}H NMR (400 MHz, $CDCl_3$) δ 7.90 – 8.00 (m, 4H), 7.06 (d, J = 8.5 Hz, 2H), 6.98 (d, J = 8.5 Hz, 2H), 4.78 (s, 2H), 4.20 (t, J = 5.9 Hz, 2H), 3.60 (t, J = 6.0 Hz, 2H), 2.56 (s, 1H), 2.35 (p, J = 6.0 Hz, 2H). ^{13}C NMR (101 MHz, $CDCl_3$) δ 193.39, 193.31, 163.99, 162.61, 132.45, 132.32, 126.97, 126.40, 115.14, 114.76, 77.41, 76.48, 65.68, 55.97, 31.98, 29.59. ESI-MS m/z: 401.0 $[M+H]^+$.

1-(4-(3-(diethylamino)propoxy)phenyl)-2-(4-(prop-2-yn-1-yloxy)phenyl)ethane-1,2-dione (4a): To a stirred suspension of **3a** (0.40 g, 1.0 mmol) and anhydrous K_2CO_3 (0.28 g, 2.0 mmol) in dry acetonitrile (20 mL), excess diethylamine (1.0 mL, 10.0 mmol) was added, and the resulting mixture was heated under reflux for 4 h until the starting material disappeared. The K_2CO_3 was removed through filtration, and the remaining solution was concentrated under reduced pressure. The crude product was purified by flash gel chromatography with CH_2Cl_2/MeOH (30:1) as elution solvents to give the desired product **4a** (0.30 g, 76% yield). ^{1}H NMR (400 MHz, DMSO-d_6) δ 7.66 – 7.88 (m, 4H), 7.10 – 7.16 (m, 4H), 4.97 (s, 2H), 4.13 (t, J = 6.1 Hz, 2H), 3.68 (s, 1H), 2.58 – 2.43 (m, 6H), 1.98 – 1.53 (m, 2H), 0.94 (t, J = 7.0 Hz, 6H). ^{13}C NMR (101 MHz, DMSO-d_6) δ 194.07, 193.87, 164.75, 163.06, 132.57, 132.40, 126.44, 125.67, 116.06, 115.64, 79.50, 78.82, 66.92, 56.43, 48.82, 46.84, 26.68, 12.11. ESI-MS m/z: 394.2 $[M+H]^+$.

7-(diethylamino)-3-(4-(4-(3-(diethylamino)propoxy)phenyl)-5-(4-(prop-2-yn-1-yloxy)phenyl)-1H-imidazol-2-yl)-2H-chromen-2-one (1): A mixture of **4a** (0.39 g, 1.0 mmol), 7-diethylaminocoumarin-3-aldehyde (0.36 g, 1.5 mmol), NH_4OAc (1.54 g, 20.0 mmol) and AcOH (8 mL) was stirred at reflux temperature for 10 hours. After cooling, the mixture was treated with 3.0 M NaOH (aq) to reach the pH of 8, and the product was exacted by CH_2Cl_2 (5 × 20 mL). The combined organic phase was dried over Na_2SO_4 and the solvent was removed under reduced pressure. The crude product was purified by using flash column chromatography with CH_2Cl_2/MeOH (20:1) as elution solvents to afford an orange solid **1** (0.40 g, 65% yield). m.p. 135-137 °C. ^{1}H NMR (400 MHz, $CDCl_3$) δ 11.05 (s, 1H), 8.71 (s, 1H), 7.65 – 7.55 (m, 2H), 7.49 – 7.35 (m, 3H), 7.07 – 6.83 (m, 4H), 6.66 (d, J = 8.9 Hz, 1H), 6.56 (s, 1H), 4.72 (s, 2H), 4.14 – 3.96 (m, 2H), 3.45 (q, J = 7.0 Hz, 4H), 2.71 – 2.64 (m, 2H), 2.65 – 2.55 (m, 4H), 2.54 (s, 1H), 2.08 – 1.89 (m, 2H), 1.25 (t, J = 7.1 Hz, 6H), 1.08 (t, J = 7.1 Hz, 6H). ^{13}C NMR (101 MHz, $CDCl_3$) δ 162.08, 156.02, 151.00, 141.41, 138.83, 129.70, 129.00, 115.21, 114.83, 109.83, 108.99, 97.06, 75.71, 75.39, 66.38, 55.89, 49.43, 47.01, 44.94, 26.90, 12.48, 11.63. Purity: 99% by HPLC. HRMS (ESI) m/z: calcd for $C_{38}H_{42}N_4O_4$: 310.1676 $[M+2H]^{2+}$. Found 310.1673 $[M+2H]^{2+}$.

General Method for 1,4-adducts synthesis via click chemistry: One mole equivalent of compound **1** (0.20 g) was dissolved in a 2:1 mixture of *t*-BuOH/H_2O (3 mL). $CuSO_4 \cdot 5H_2O$ (100 µL, 100 mM) and sodium ascorbate (300 µL, 100 mM) were added and the solution stirred for 10 min. The respective azide (5 mole equivalent) was added and the solution was allowed to stir overnight under 80 °C. The solvent was removed under reduced pressure and the products were purified by using flash column chromatography with CH_2Cl_2/MeOH (20:1) as elution solvents.

7-(diethylamino)-3-(4-(4-(3-(diethylamino)propoxy)phenyl)-5-(4-((1-(2-(piperidin-1-yl)ethyl)-1H-1,2,3-triazol-4-yl)methoxy)phenyl)-1H-imidazol-2-yl)-2H-chromen-2-one (9): Orange solid (0.15 g, 62% yield). m.p.

119-121 °C. ^{1}H NMR (400 MHz, $CDCl_3$) δ 11.06 (s, 1H), 8.71 (s, 1H), 7.80 (s, 1H), 7.68 – 7.32 (m, 5H), 7.08 – 6.79 (m, 4H), 6.67 (d, *J* = 11.3 Hz, 1H), 6.57 (s, 1H), 5.25 (s, 2H), 4.47 (t, *J* = 6.4 Hz, 2H), 4.06 (t, *J* = 5.8 Hz, 2H), 3.46 (q, *J* = 7.0 Hz, 4H), 2.78 (t, *J* = 6.4 Hz, 2H), 2.74 – 2.67 (m, 2H), 2.63 (q, *J* = 7.1 Hz, 4H), 2.49 – 2.40 (m, 4H), 2.08 – 1.93 (m, 2H), 1.66 – 1.51 (m, 4H), 1.52 – 1.39 (m, 2H), 1.25 (t, *J* = 7.1 Hz, 6H), 1.09 (t, *J* = 7.2 Hz, 6H). ^{13}C NMR (101 MHz, $CDCl_3$) δ 161.97, 155.93, 150.98, 141.33, 138.84, 129.72, 128.93, 123.52, 114.86, 114.58, 109.83, 108.86, 96.89, 66.20, 62.03, 58.14, 54.44, 49.30, 47.81, 46.86, 44.87, 26.55, 25.88, 24.08, 12.44, 11.34. Purity: 98% by HPLC. HRMS (ESI) m/z: calcd for $C_{45}H_{56}N_8O_4$: 387.2285 $[M+2H]^{2+}$. Found 387.2278 $[M+2H]^{2+}$.

7-(diethylamino)-3-(4-(4-(3-(diethylamino)propoxy)phenyl)-5-(4-((1-(3-(4-methylpiperazin-1-yl)propyl)-1H-1,2,3-triazol-4-yl)methoxy)phenyl)-1H-imidazol-2-yl)-2H-chromen-2-one (10): Orange solid (0.20 g, 77% yield). m.p. 125-128 °C. ^{1}H NMR (400 MHz, $CDCl_3$) δ 11.05 (s, 1H), 8.70 (s, 1H), 7.68 – 7.32 (m, 6H), 7.04 – 6.81 (m, 4H), 6.66 (d, *J* = 6.8 Hz, 1H), 6.55 (s, 1H), 5.24 (s, 2H), 4.43 (t, *J* = 6.0 Hz, 2H), 4.00 – 4.04 (m, 2H), 3.44 (q, *J* = 6.9 Hz, 4H), 2.69 – 2.63 (m, 2H), 2.58 (q, *J* = 7.2 Hz, 4H), 2.51 – 2.38 (m, 8H), 2.33 (t, *J* = 6.7 Hz, 2H), 2.27 (s, 3H), 2.12 – 2.04 (m, 2H), 2.01 – 1.91 (m, 2H), 1.24 (t, *J* = 7.1 Hz, 6H), 1.06 (t, *J* = 7.1 Hz, 6H). ^{13}C NMR (101 MHz, $CDCl_3$) δ 162.06, 156.01, 151.01, 141.34, 138.82, 129.70, 128.98, 122.99, 114.83, 114.70, 109.83, 108.97, 97.05, 66.36, 62.18, 55.08, 54.41, 52.95, 49.43, 48.20, 47.00, 45.97, 44.92, 27.27, 26.90, 12.46, 11.63. Purity: 99% by HPLC. HRMS (ESI) m/z: calcd for $C_{46}H_{59}N_9O_4$: 401.7418 $[M+2H]^{2+}$. Found 401.7411 $[M+2H]^{2+}$.

7-(diethylamino)-3-(4-(4-(3-(diethylamino)propoxy)phenyl)-5-(4-((1-(2-morpholinoethyl)-1H-1,2,3-triazol-4-yl)methoxy)phenyl)-1H-imidazol-2-yl)-2H-chromen-2-one (11): Orange solid (0.21 g, 84% yield). m.p. 126-129 °C. ^{1}H NMR (400 MHz, $CDCl_3$) δ 11.04 (s, 1H), 8.70 (s, 1H), 7.77 (d, *J* = 8.6 Hz, 1H), 7.66 – 7.33 (m, 5H), 7.03 – 6.80 (m, 4H), 6.66 (d, *J* = 8.6 Hz, 1H), 6.56 (s, 1H), 5.25 (s, 2H), 4.57 – 4.38 (m, 2H), 4.10 – 3.99 (m, 2H), 3.75 – 3.62 (m, 4H), 3.53 – 3.38 (m, 4H), 2.93 – 2.34 (m, 12H), 2.14 – 1.91 (m, 2H), 1.24 (t, *J* = 6.5 Hz, 6H), 1.20 – 1.01 (m, 6H). ^{13}C NMR (101 MHz, $CDCl_3$) δ 161.97, 155.96, 150.99, 141.34, 138.79, 129.69, 128.94, 123.35, 115.02, 114.78, 109.83, 108.89, 96.95, 66.78, 66.30, 62.05, 57.78, 53.43, 49.36, 47.43, 46.93, 44.88, 26.78, 12.45, 11.55. Purity: 97% by HPLC. HRMS (ESI) m/z: calcd for $C_{44}H_{54}N_8O_5$: 388.2181 $[M+2H]^{2+}$. Found 388.2168 $[M+2H]^{2+}$.

7-(diethylamino)-3-(4-(4-(3-(diethylamino)propoxy)phenyl)-5-(4-((1-(2-(dimethylamino)ethyl)-1H-1,2,3-triazol-4-yl)methoxy)phenyl)-1H-imidazol-2-yl)-2H-chromen-2-one (12): Orange solid (0.14 g, 62% yield). m.p. 105-107 °C. ^{1}H NMR (400 MHz, $CDCl_3$) δ 11.05 (s, 1H), 8.70 (s, 1H), 7.77 (s, 1H), 7.66 – 7.33 (m, 5H), 7.07 – 6.81 (m, 4H), 6.66 (d, *J* = 8.9 Hz, 1H), 6.55 (s, 1H), 5.23 (s, 2H), 4.46 (t, *J* = 6.2 Hz, 2H), 4.04 (t, *J* = 6.2 Hz, 2H), 3.44 (q, *J* = 7.0 Hz, 4H), 2.78 (t, *J* = 6.3 Hz, 2H), 2.71 – 2.64 (m, 2H), 2.60 (q, *J* = 7.1 Hz, 4H), 2.29 (s, 6H), 2.02 – 1.91 (m, 2H), 1.23 (t, *J* = 7.0 Hz, 6H), 1.07 (t, *J* = 7.1 Hz, 6H). ^{13}C NMR (101 MHz, $CDCl_3$) δ 162.05, 156.01, 151.01,

141.35, 138.82, 129.70, 128.98, 123.30, 114.76, 114.45, 109.85, 108.97, 97.04, 66.34, 62.18, 58.71, 49.40, 48.25, 46.97, 45.35, 44.92, 26.82, 12.47, 11.56. Purity: 98% by HPLC. HRMS (ESI) m/z: calcd for $C_{42}H_{52}N_8O_4$: 733.4184 $[M+H]^+$. Found 733.4150 $[M+H]^+$.

7-(diethylamino)-3-(4-(4-(3-(diethylamino)propoxy)phenyl)-5-(4-((1-(3-(dimethylamino)propyl)-1H-1,2,3-triazol-4-yl)methoxy)phenyl)-1H-imidazol-2-yl)-2H-chromen-2-one (13): Orange solid (0.13 g, 56% yield). m.p. 107-109 °C. ^{1}H NMR (400 MHz, $CDCl_3$) δ 11.05 (s, 1H), 8.70 (s, 1H), 7.78 – 7.32 (m, 6H), 7.08 – 6.78 (m, 4H), 6.65 (d, *J* = 10.7 Hz, 1H), 6.55 (s, 1H), 5.24 (s, 2H), 4.44 (t, *J* = 6.7 Hz, 2H), 4.04 (t, *J* = 5.7 Hz, 2H), 3.44 (q, *J* = 6.9 Hz, 4H), 2.72 – 2.63 (m, 2H), 2.59 (q, *J* = 7.1 Hz, 4H), 2.32 – 2.14 (m, 8H), 2.12 – 2.03 (m, 2H), 2.01 – 1.92 (m, 2H), 1.23 (t, *J* = 7.0 Hz, 6H), 1.07 (t, *J* = 7.1 Hz, 6H). ^{13}C NMR (101 MHz, $CDCl_3$) δ 162.13, 156.04, 151.00, 141.39, 138.88, 129.75, 129.02, 123.03, 114.79, 114.68, 109.84, 108.96, 97.02, 66.28, 62.15, 55.77, 49.40, 48.15, 46.95, 45.34, 44.98, 28.15, 26.62, 12.49, 11.41. Purity: 97% by HPLC. HRMS (ESI) m/z: calcd for $C_{43}H_{54}N_8O_4$: 374.2207 $[M+2H]^{2+}$. Found 374.2198 $[M+2H]^{2+}$.

7-(diethylamino)-3-(4-(4-(3-(diethylamino)propoxy)phenyl)-5-(4-((1-(3-hydroxypropyl)-1H-1,2,3-triazol-4-yl)methoxy)phenyl)-1H-imidazol-2-yl)-2H-chromen-2-one (14): Orange solid (0.18 g, 77% yield). m.p. 121-123 °C. ^{1}H NMR (400 MHz, $CDCl_3$) δ 11.06 (s, 1H), 8.68 (s, 1H), 7.62 – 7.33 (m, 6H), 7.04 – 6.80 (m, 4H), 6.65 (d, *J* = 6.6 Hz, 1H), 6.54 (s, 1H), 5.22 (s, 2H), 4.56 – 4.38 (m, 2H), 4.03 (t, *J* = 6.0 Hz, 2H), 3.65 – 3.51 (m, 2H), 3.44 (q, *J* = 6.9 Hz, 4H), 2.68 – 2.62 (m, 2H), 2.58 (q, *J* = 7.1 Hz, 4H), 2.16 – 2.02 (m, 2H), 2.00 – 1.89 (m, 2H), 1.23 (t, *J* = 7.1 Hz, 6H), 1.05 (t, *J* = 7.1 Hz, 6H). ^{13}C NMR (101 MHz, $CDCl_3$) δ 162.11, 156.03, 151.04, 141.41, 138.94, 129.79, 129.03, 123.34, 115.12, 114.80, 109.86, 108.94, 97.00, 66.16, 62.10, 58.40, 49.35, 47.06, 46.89, 44.98, 32.63, 26.30, 12.49, 11.11. Purity: 98% by HPLC. HRMS (ESI) m/z: calcd for $C_{41}H_{49}N_7O_5$: 720.3868 $[M+H]^+$. Found 720.3830 $[M+H]^+$.

3-(4-((4-(2-(7-(diethylamino)-2-oxo-2H-chromen-3-yl)-4-(4-(3-(diethylamino)propoxy)phenyl)-1H-imidazol-5-yl)phenoxy)methyl)-1H-1,2,3-triazol-1-yl)propanoic acid (15): Orange solid (0.07 g, 31% yield). m.p. 122-125 °C. ^{1}H NMR (400 MHz, CD_3OD) δ 8.25 (s, 1H), 7.97 (s, 1H), 7.39 – 7.06 (m, 5H), 6.89 – 6.69 (m, 4H), 6.61 (d, *J* = 6.9 Hz, 1H), 6.37 (s, 1H), 4.98 (s, 2H), 4.50 – 4.57 (m, 2H), 4.06 – 3.82 (m, 2H), 3.53 (s, 1H), 3.42 – 3.27 (m, 4H), 3.20 – 3.04 (m, 6H), 2.69 – 2.76 (m, 2H), 2.20 – 1.91 (m, 2H), 1.23 (t, *J* = 6.8 Hz, 6H), 1.10 (t, *J* = 6.4 Hz, 6H). ^{13}C NMR (101 MHz, CD_3OD) δ 161.27, 157.87, 155.84, 151.37, 141.10, 139.25, 129.84, 128.97, 125.25, 114.42, 114.18, 109.86, 108.42, 96.18, 69.88, 64.59, 61.17, 48.72, 46.77, 44.46, 29.37, 23.53, 11.59, 7.81. Purity: 97% by HPLC. HRMS (ESI) m/z: calcd for $C_{41}H_{47}N_7O_6$: 734.3661 $[M+H]^+$. Found 734.3671 $[M+H]^+$.

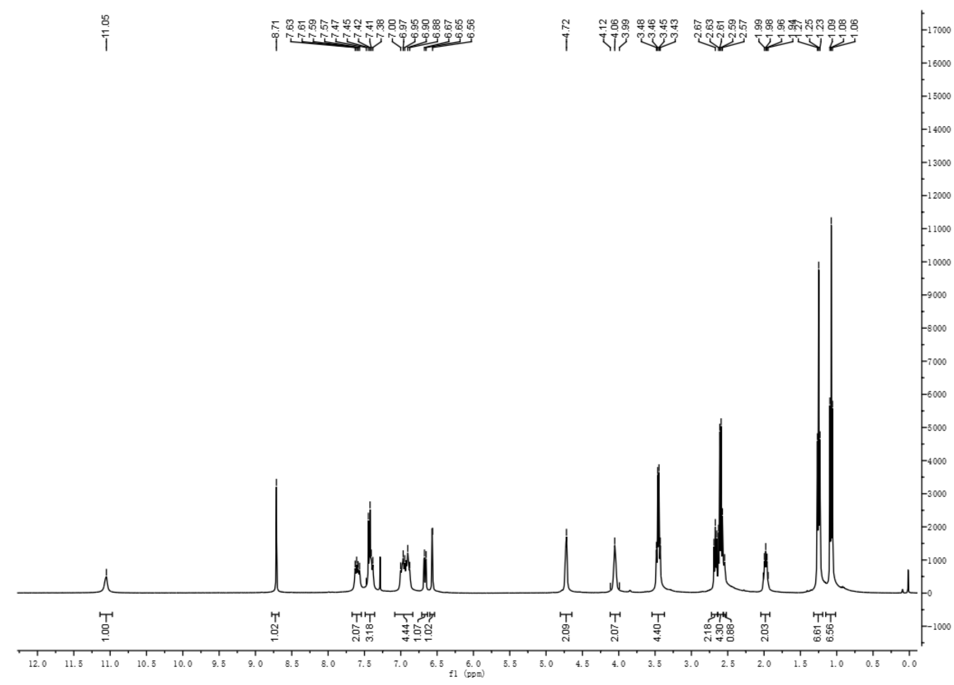

Figure S1. ^{1}H NMR spectrum of **1**

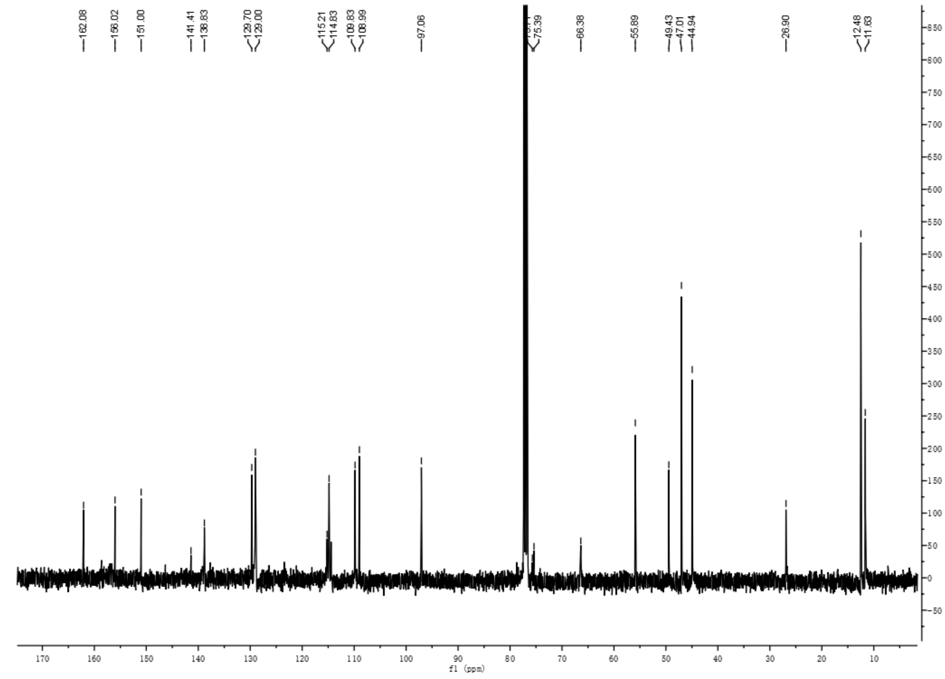

Figure S2. ^{13}C NMR spectrum of **1**

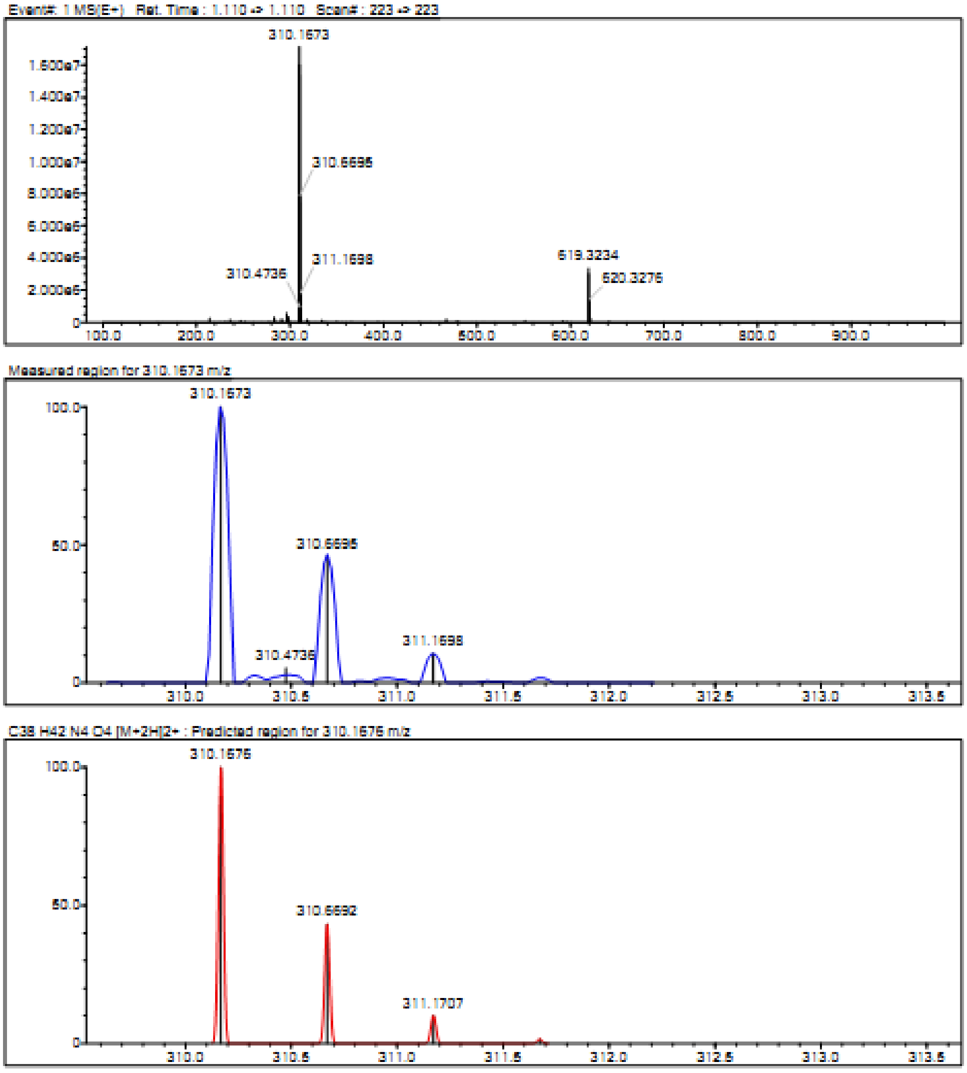

Rank	Score	Formula (M)	Ion	Meas. m/z	Pred. m/z	Df. (mDa)	Df. (ppm)	Iso	DBE
1	85.97	C38 H42 N4 O4	[M+2H]2+	310.1673	310.1676	-0.3	-0.97	85.97	20.0

Figure S3. HRMS spectrum of **1**

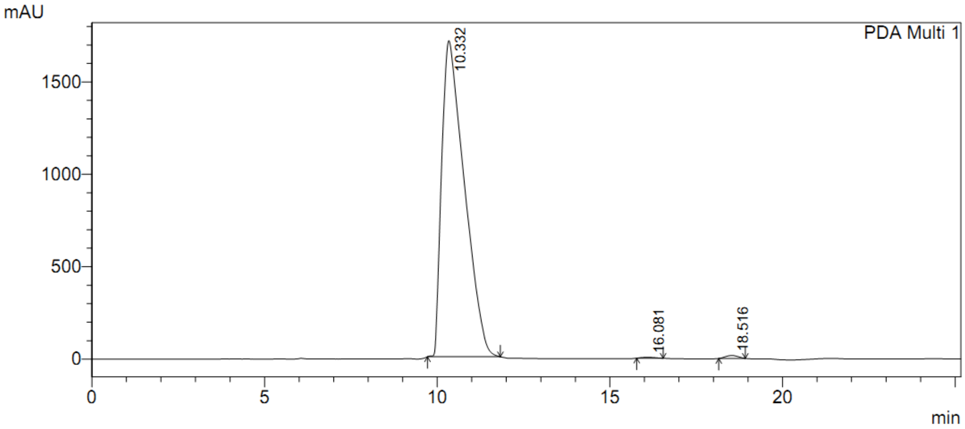

Figure S4. HPLC analysis of **1**

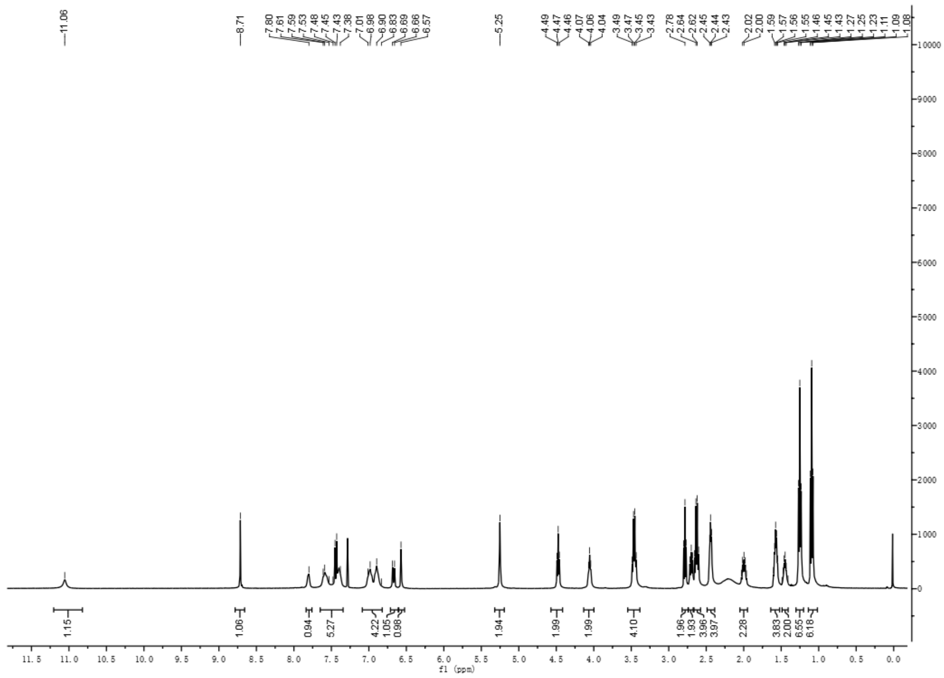

Figure S5. ^{1}H NMR spectrum of **9**

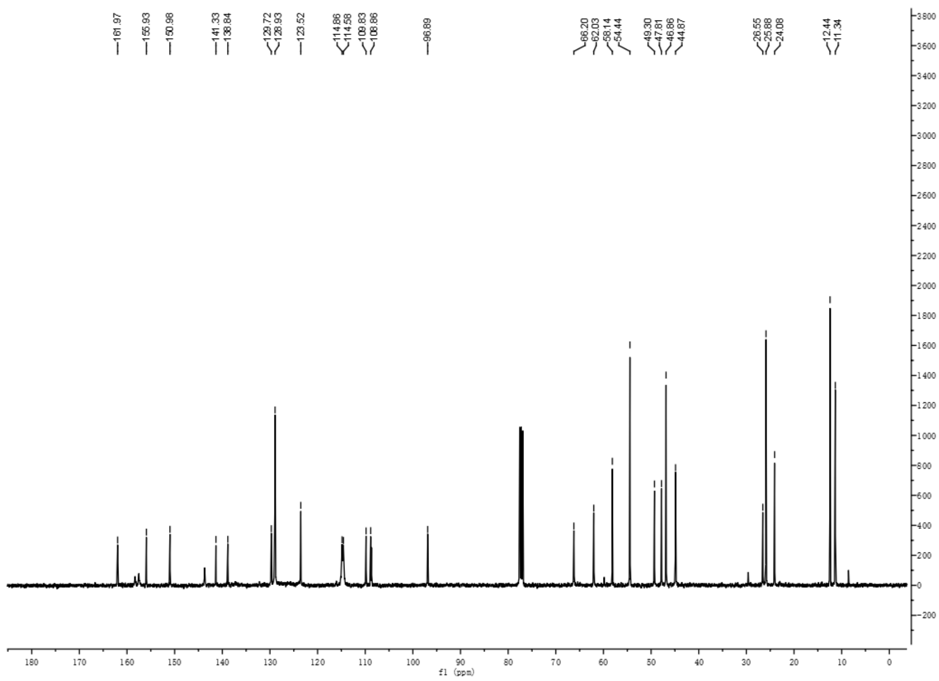

Figure S6. ^{13}C NMR spectrum of **9**

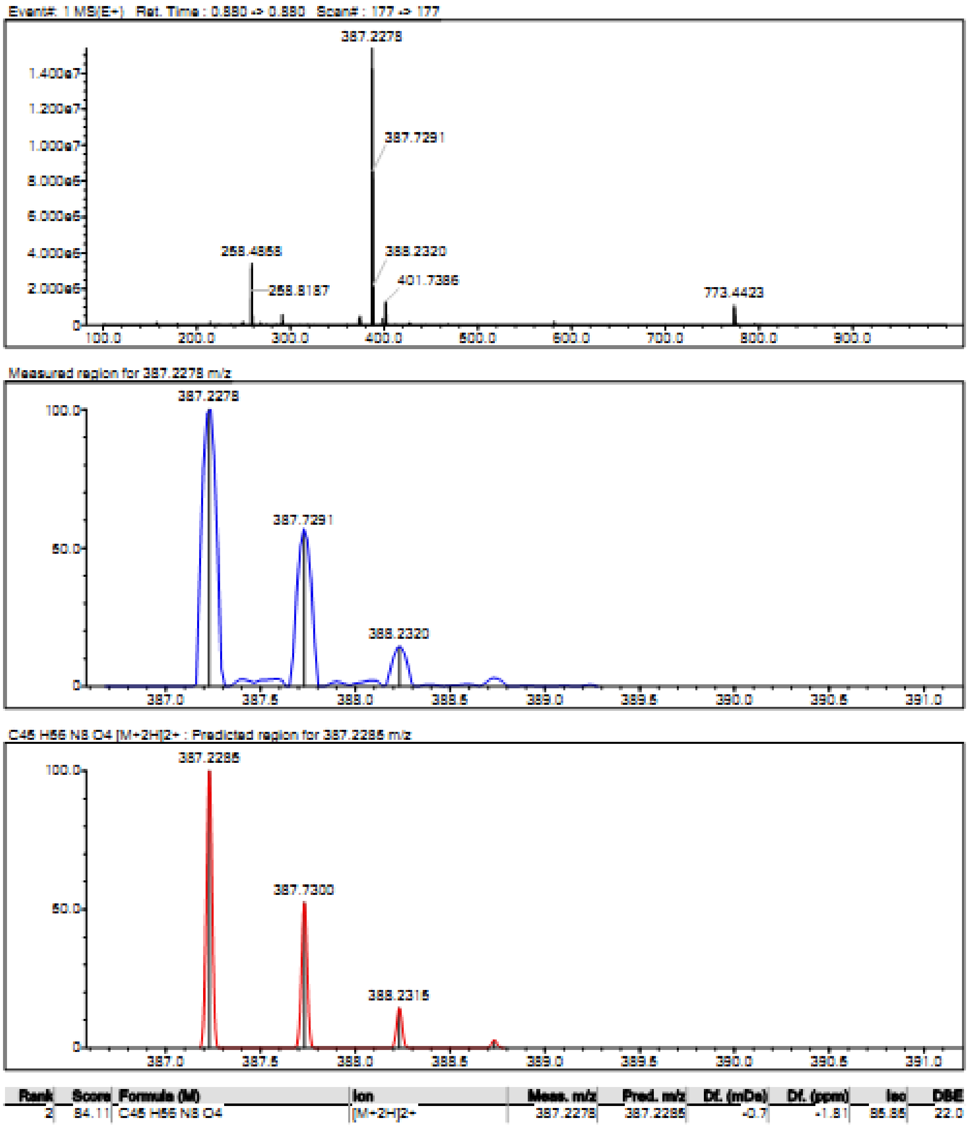

Figure S7. HRMS spectrum of **9**

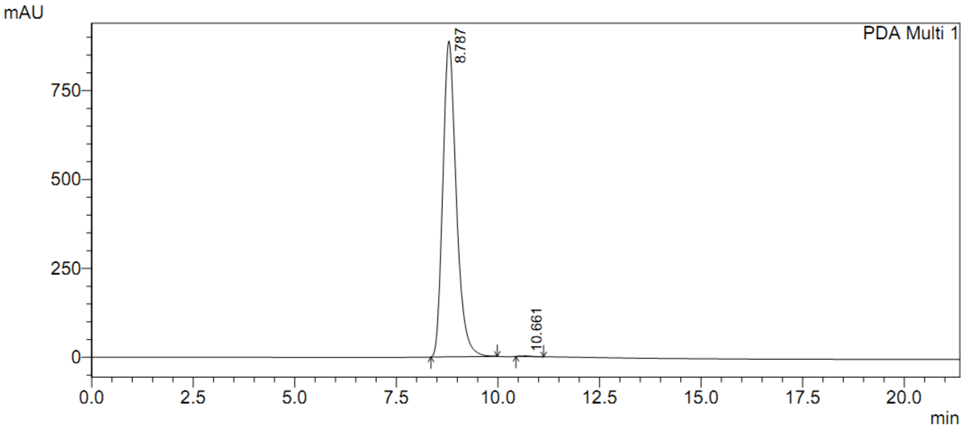

Figure S8. HPLC analysis of **9**

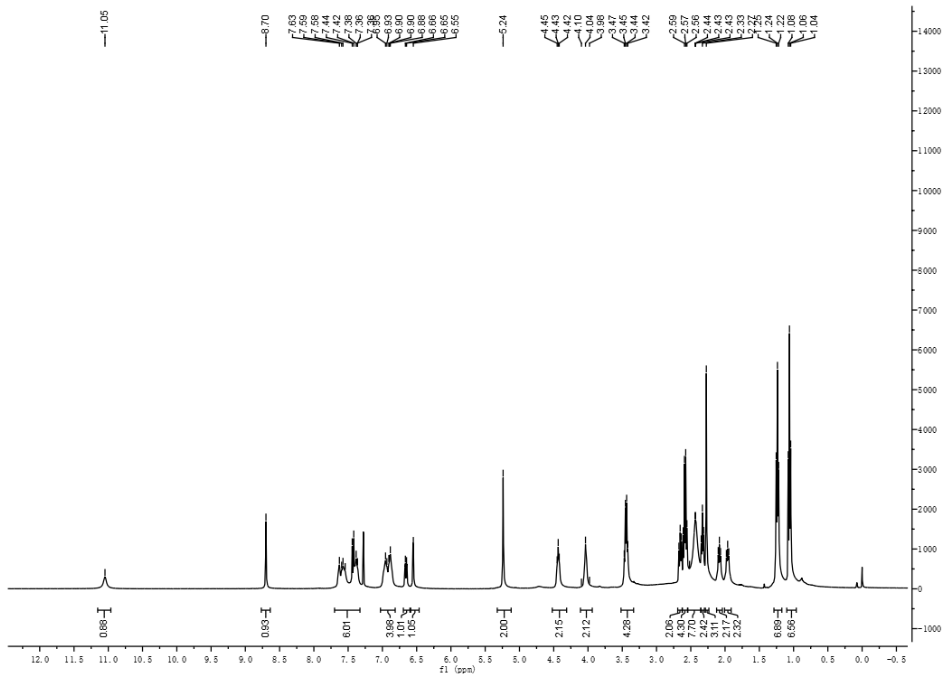

Figure S9. ^{1}H NMR spectrum of **10**

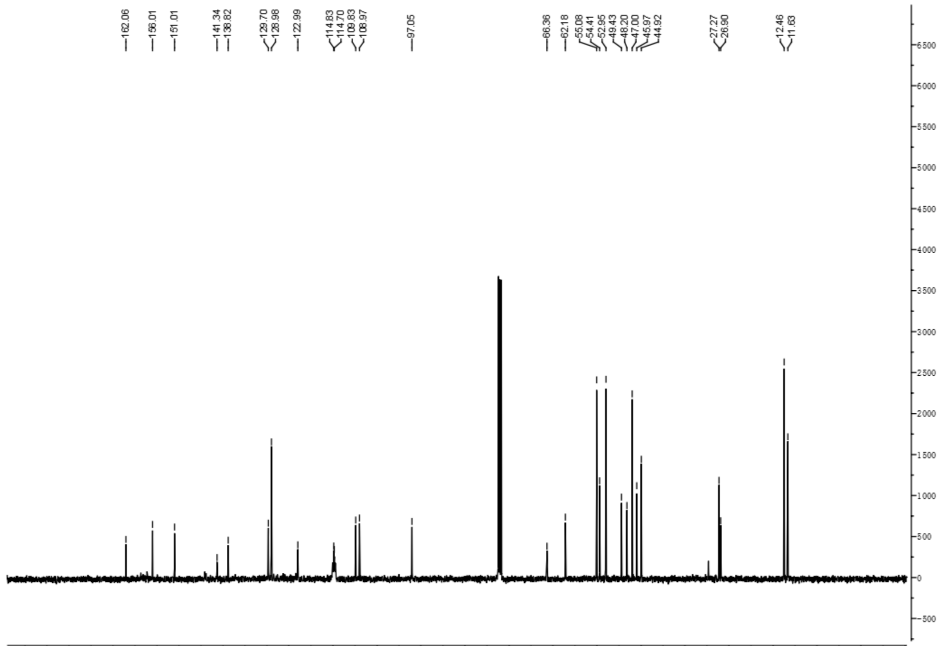

Figure S10. ^{13}C NMR spectrum of **10**

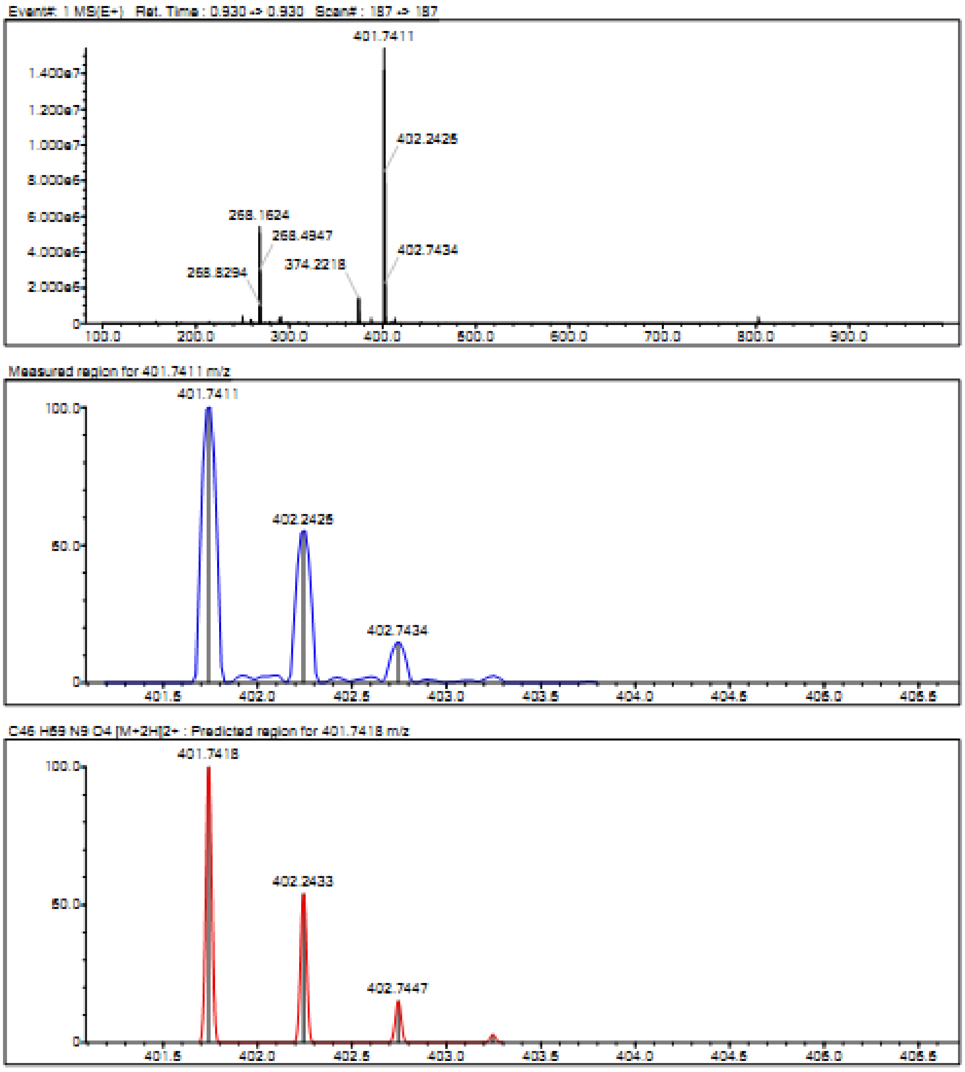

Rank	Score	Formula (M)	Ion	Meas. m/z	Pred. m/z	Df. (mDa)	Df. (ppm)	Iso	DBE
1	98.15	C46 H59 N9 O4	[M+2H]2+	401.7411	401.7418	-0.7	-1.74	100.00	22.0

Figure S11. HRMS spectrum of **10**

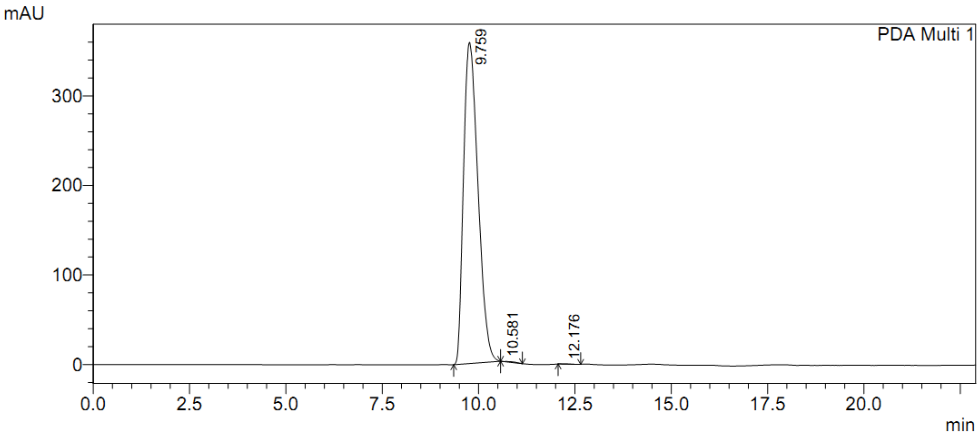

Figure S12. HPLC analysis of **10**

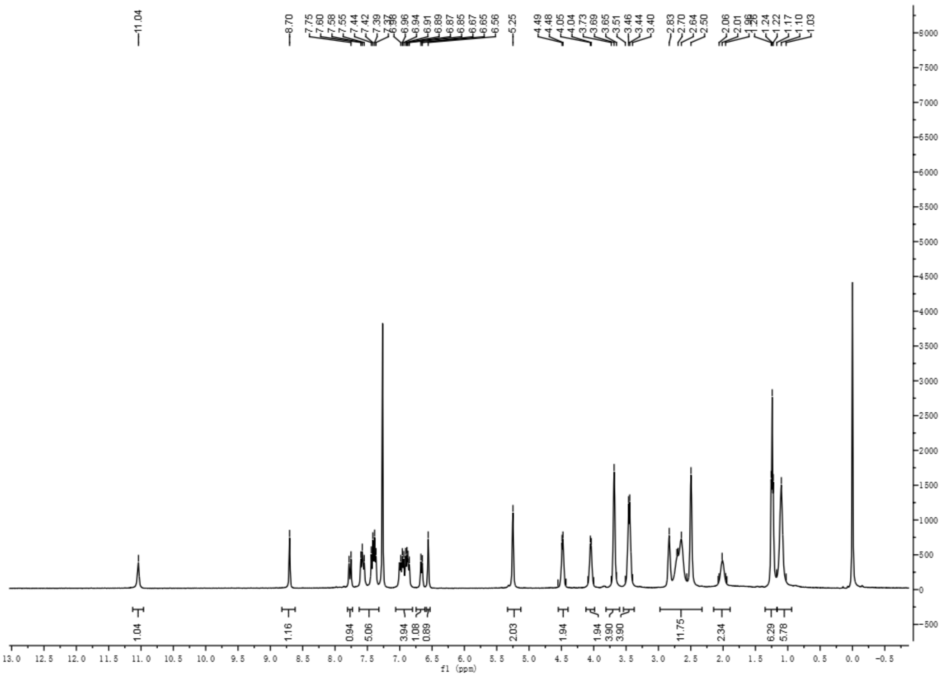

Figure S13. ^{1}H NMR spectrum of **11**

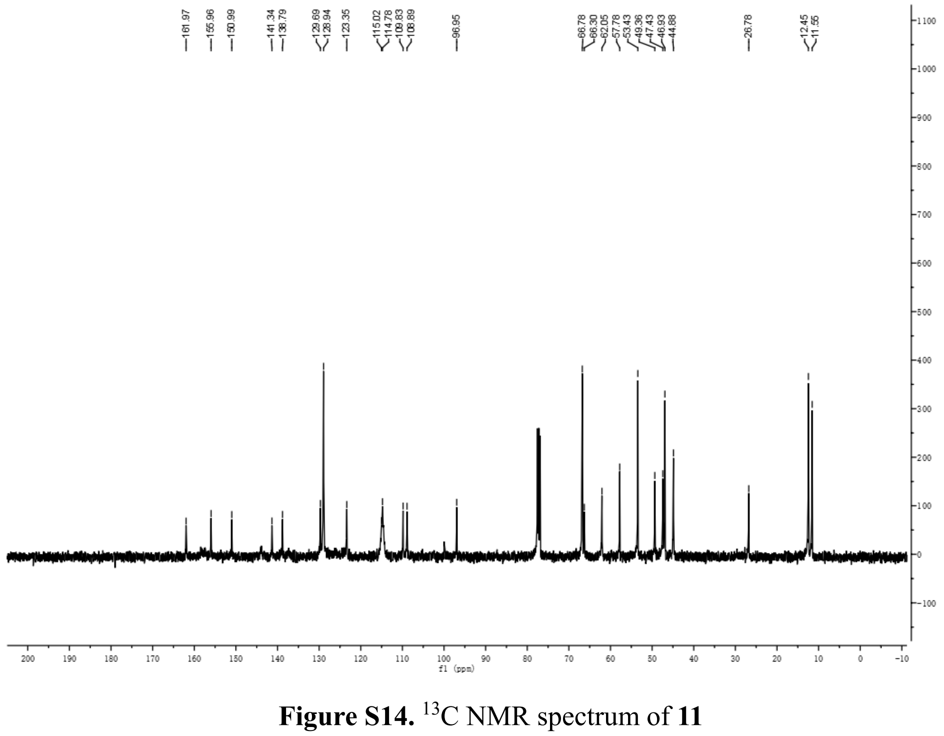

Figure S14. ^{13}C NMR spectrum of **11**

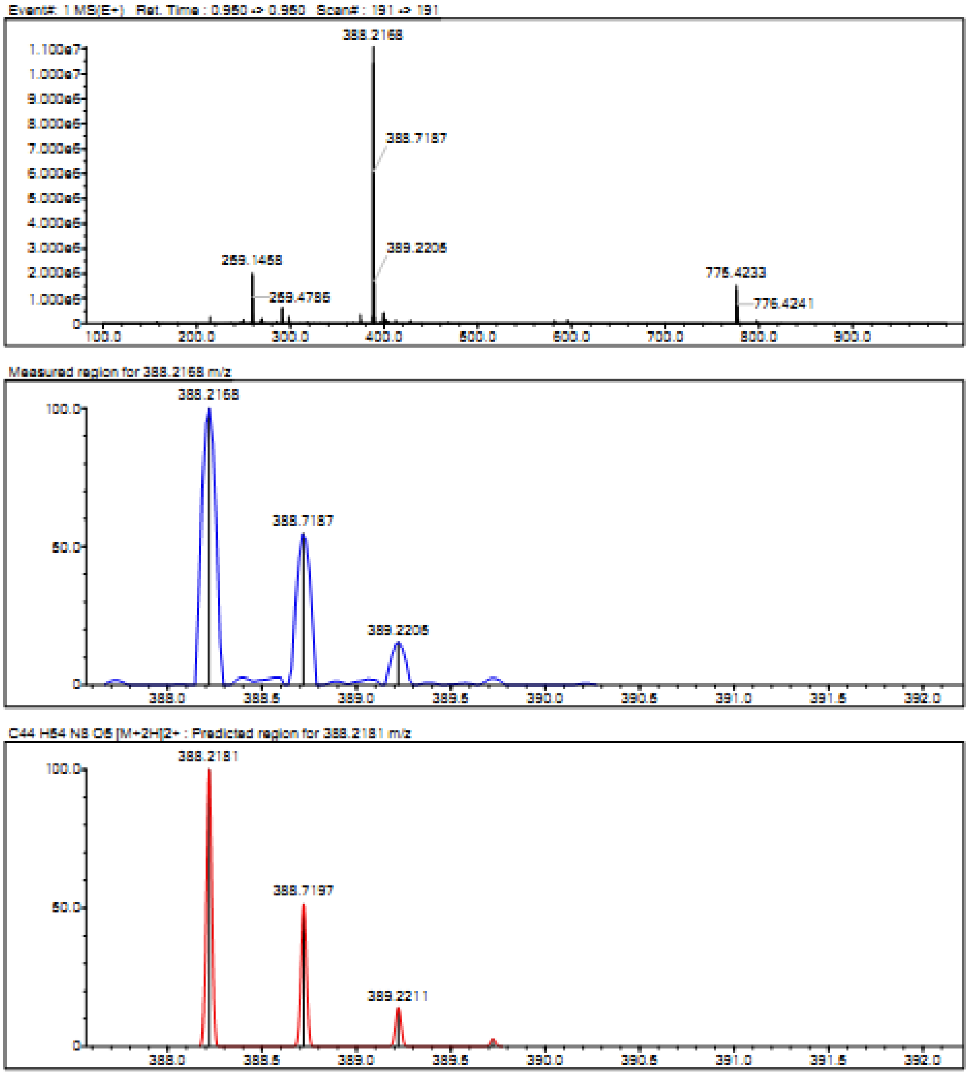

Rank	Score	Formula (M)	Ion	Meas. m/z	Pred. m/z	Df. (mDa)	Df. (ppm)	Iso	DBE
2	79.48	C44 H54 N8 O5	[M+2H]2+	388.2168	388.2181	-1.3	-3.35	84.44	22.0

Figure S15. HRMS spectrum of **11**

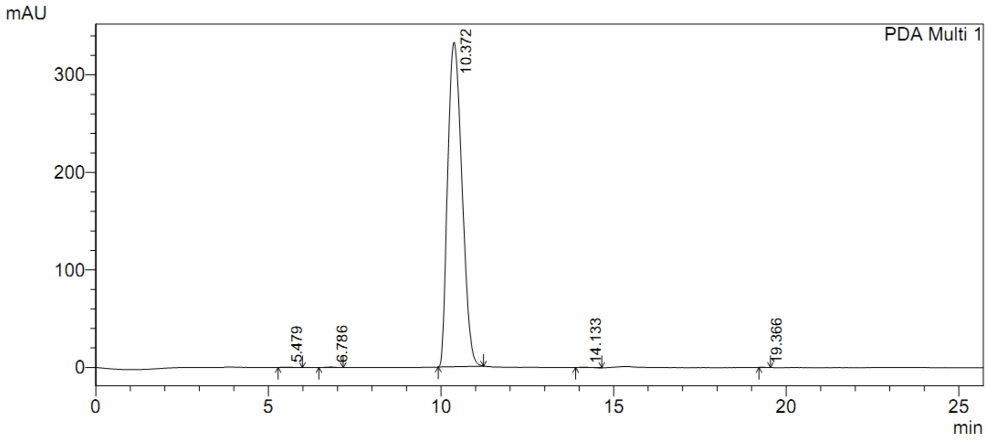

Figure S16. HPLC analysis of **11**

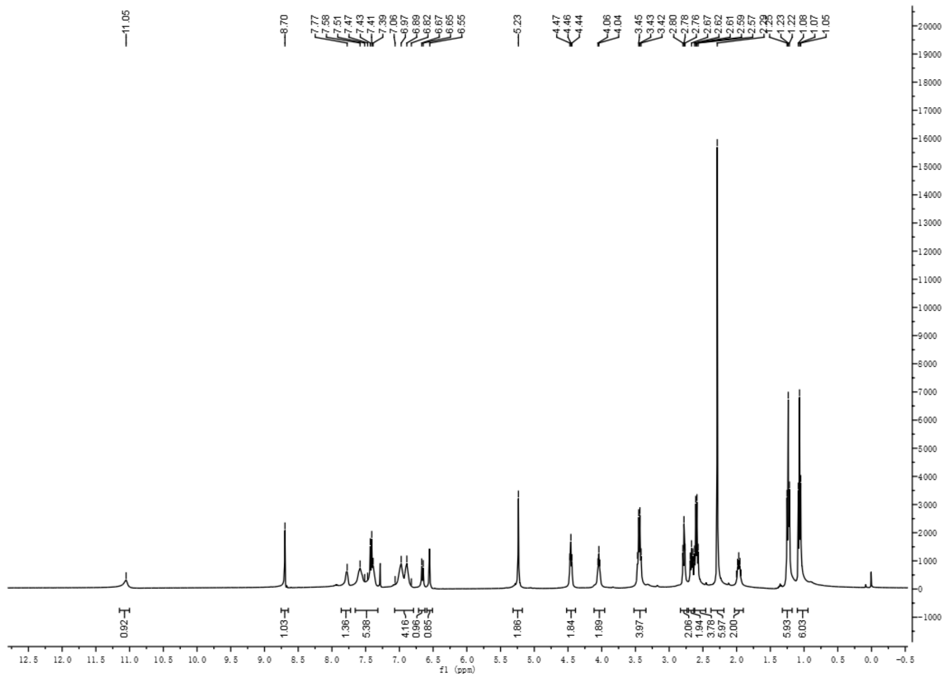

Figure S17. ^{1}H NMR spectrum of **12**

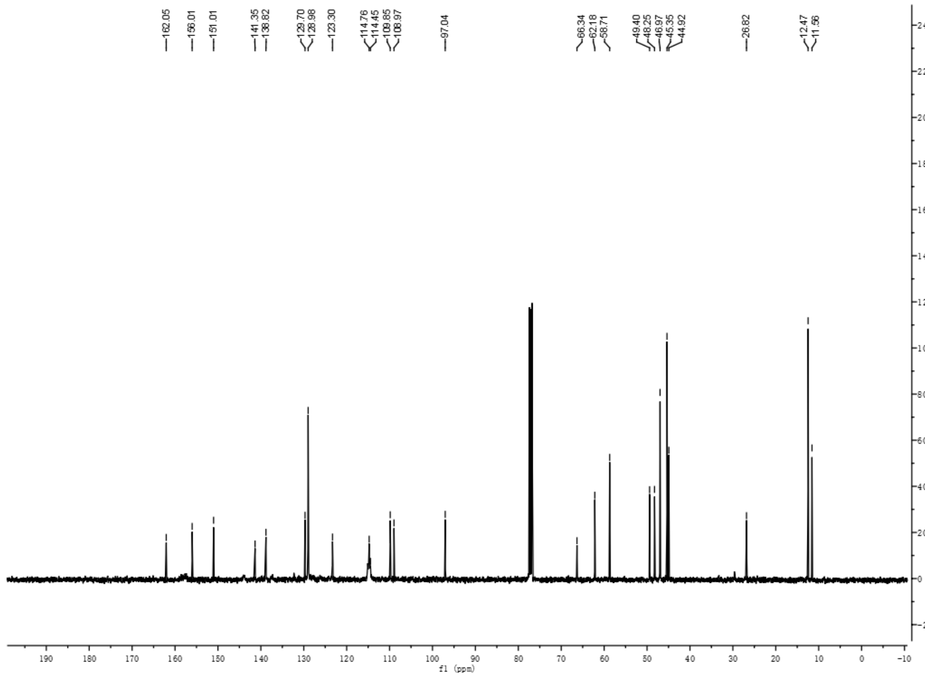

Figure S18. ^{13}C NMR spectrum of **12**

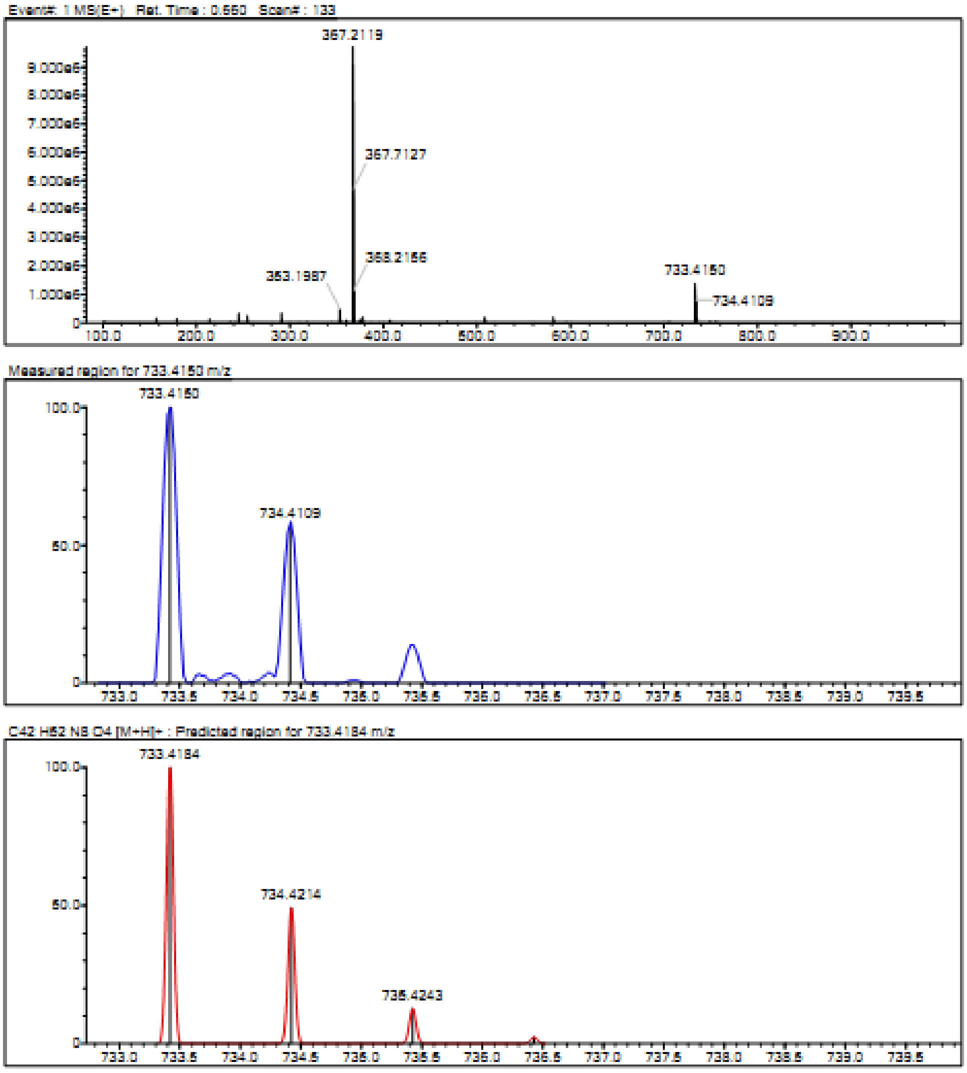

Rank	Score	Formula (M)	Ion	Meas. m/z	Pred. m/z	Df. (mDa)	Df. (ppm)	Iso	DBE
1	65.28	C42 H52 N8 O4	[M+H]+	733.4150	733.4184	-3.4	-4.64	71.82	21.0

Figure S19. HRMS spectrum of **12**

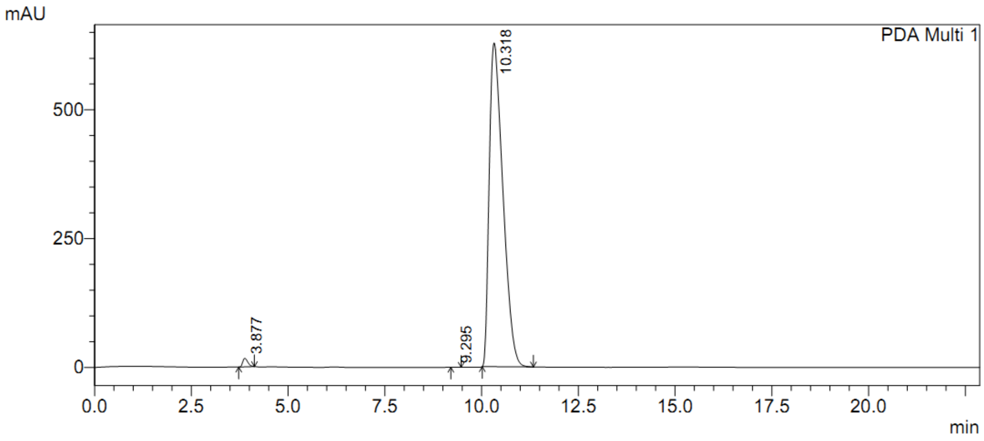

Figure S20. HPLC analysis of **12**

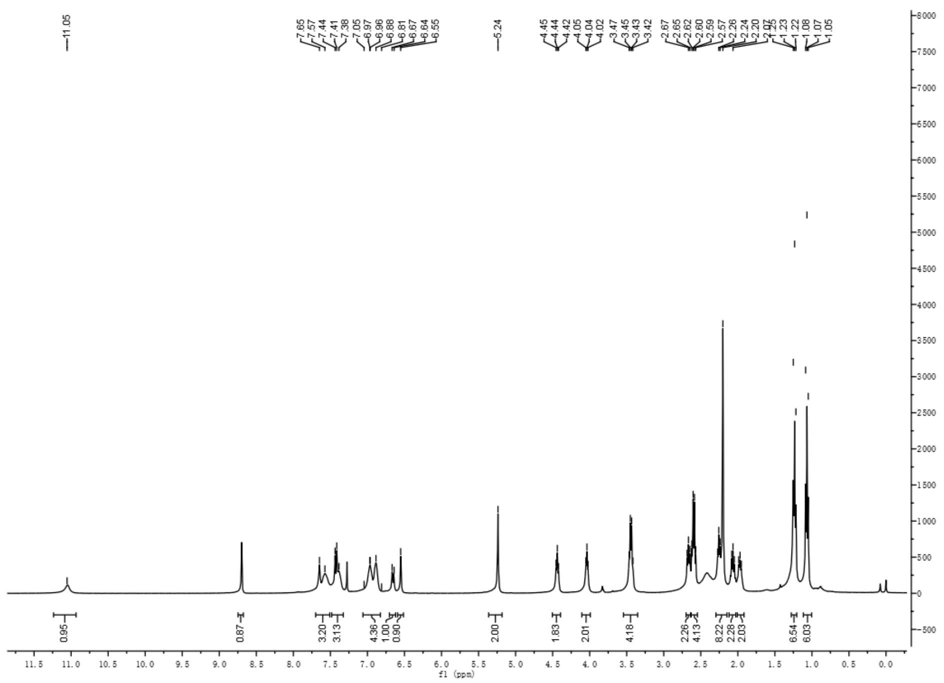

Figure S21. ^{1}H NMR spectrum of **13**

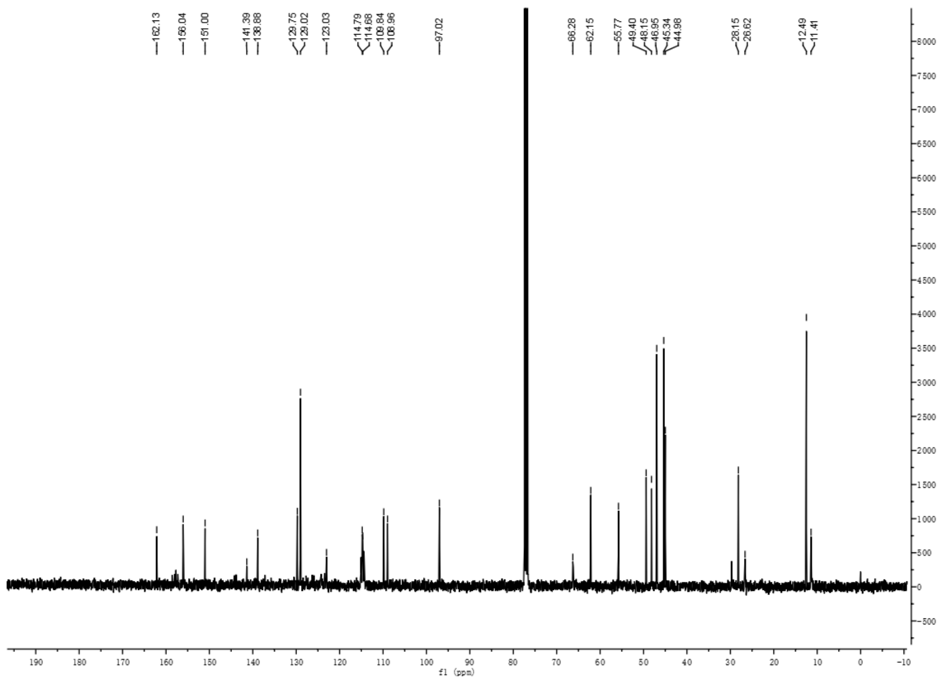

Figure S22. ^{13}C NMR spectrum of **13**

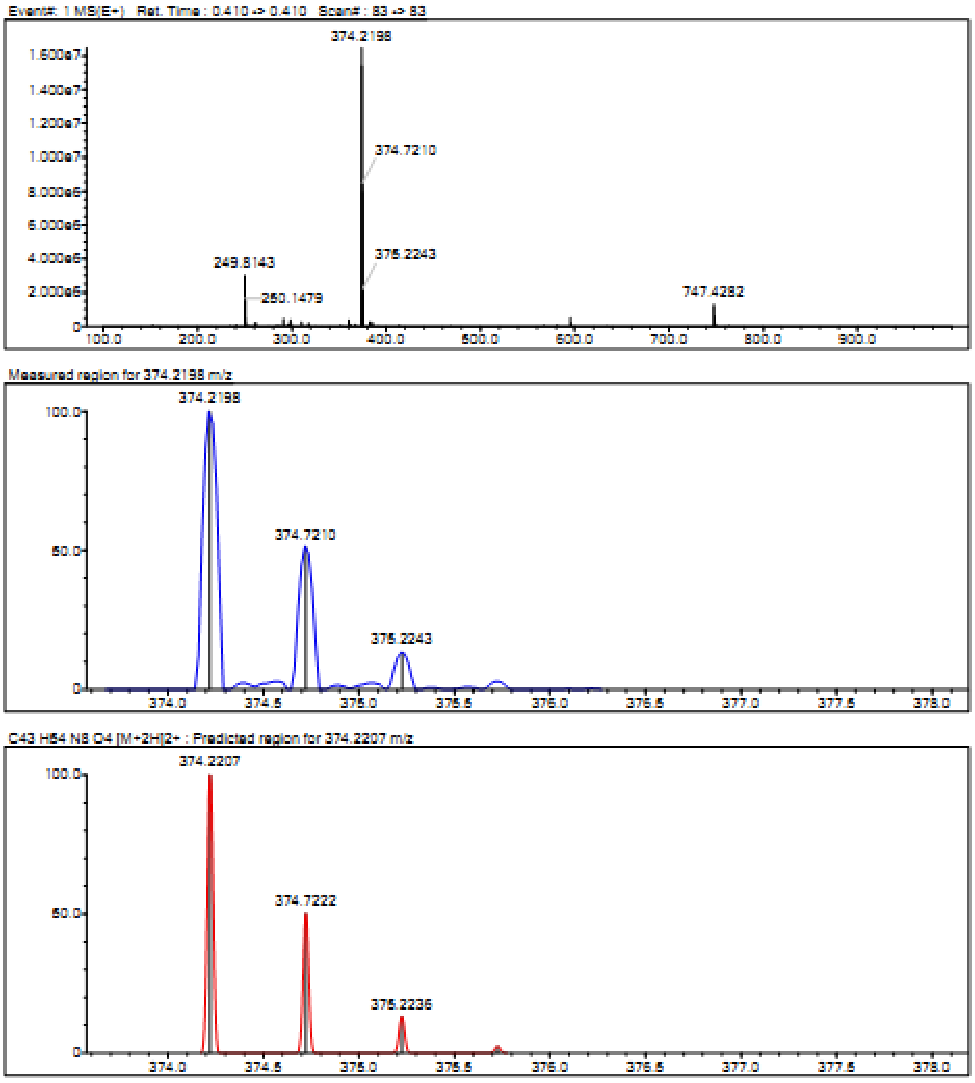

Rank	Score	Formula (M)	Ion	Meas. m/z	Pred. m/z	Df. (mDa)	Df. (ppm)	Iso	DBE
1	82.07	C43 H54 N8 O4	[M+2H]2+	374.2198	374.2207	-0.9	-2.41	85.07	21.0

Figure S23. HRMS spectrum of **13**

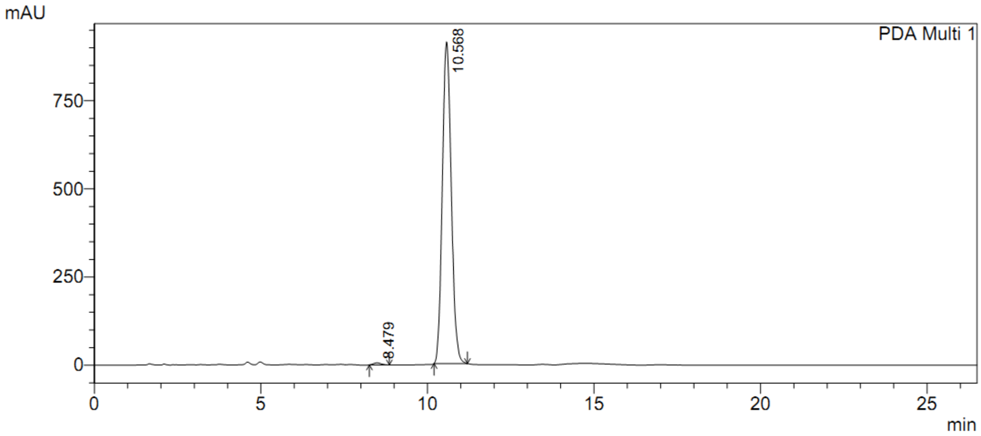

Figure S24. HPLC analysis of **13**

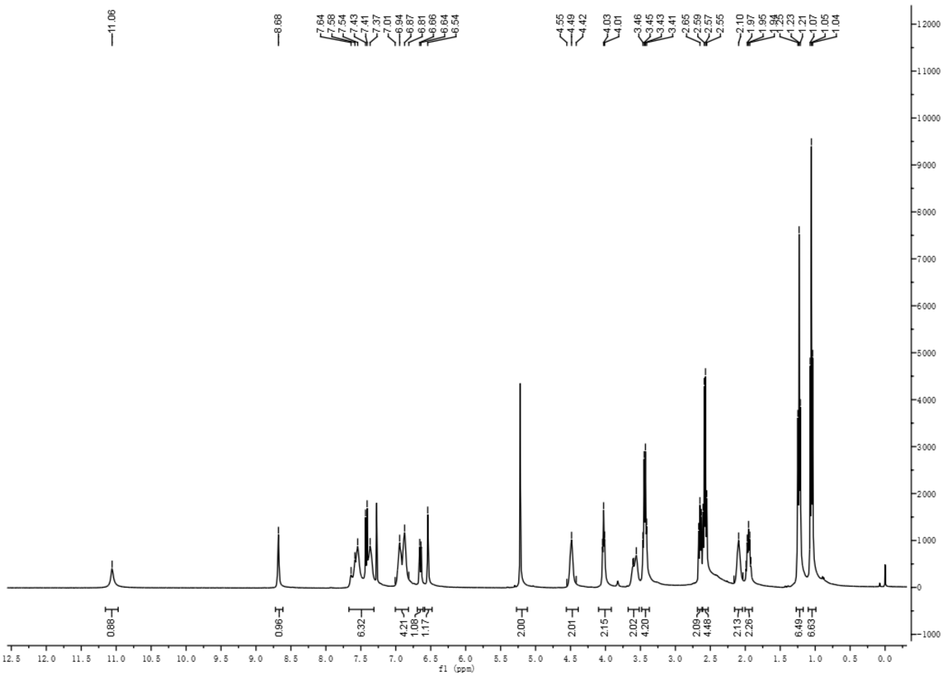

Figure S25. ^{1}H NMR spectrum of **14**

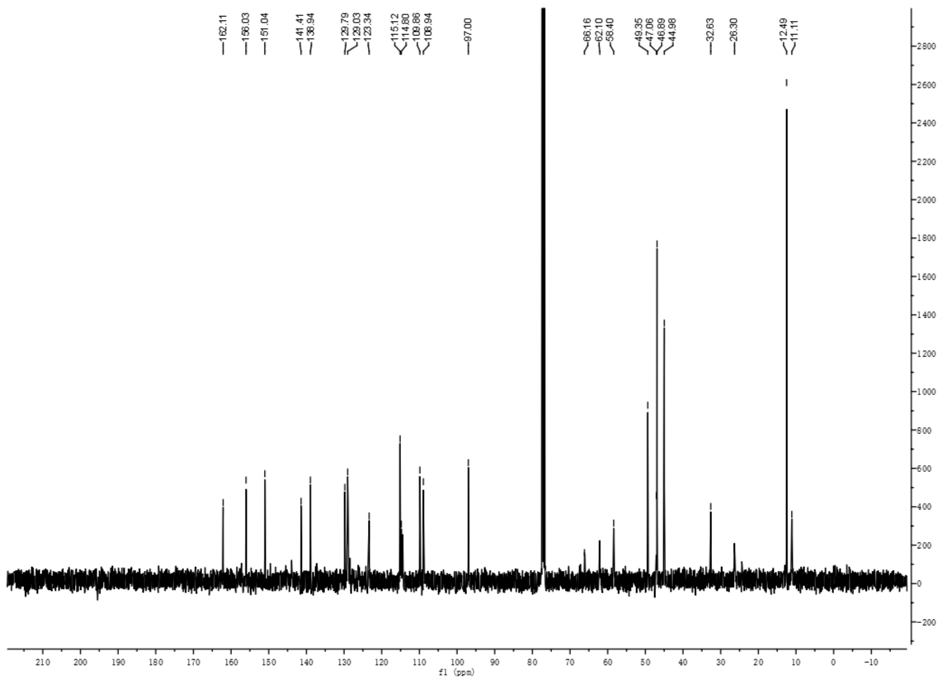

Figure S26. ^{13}C NMR spectrum of **14**

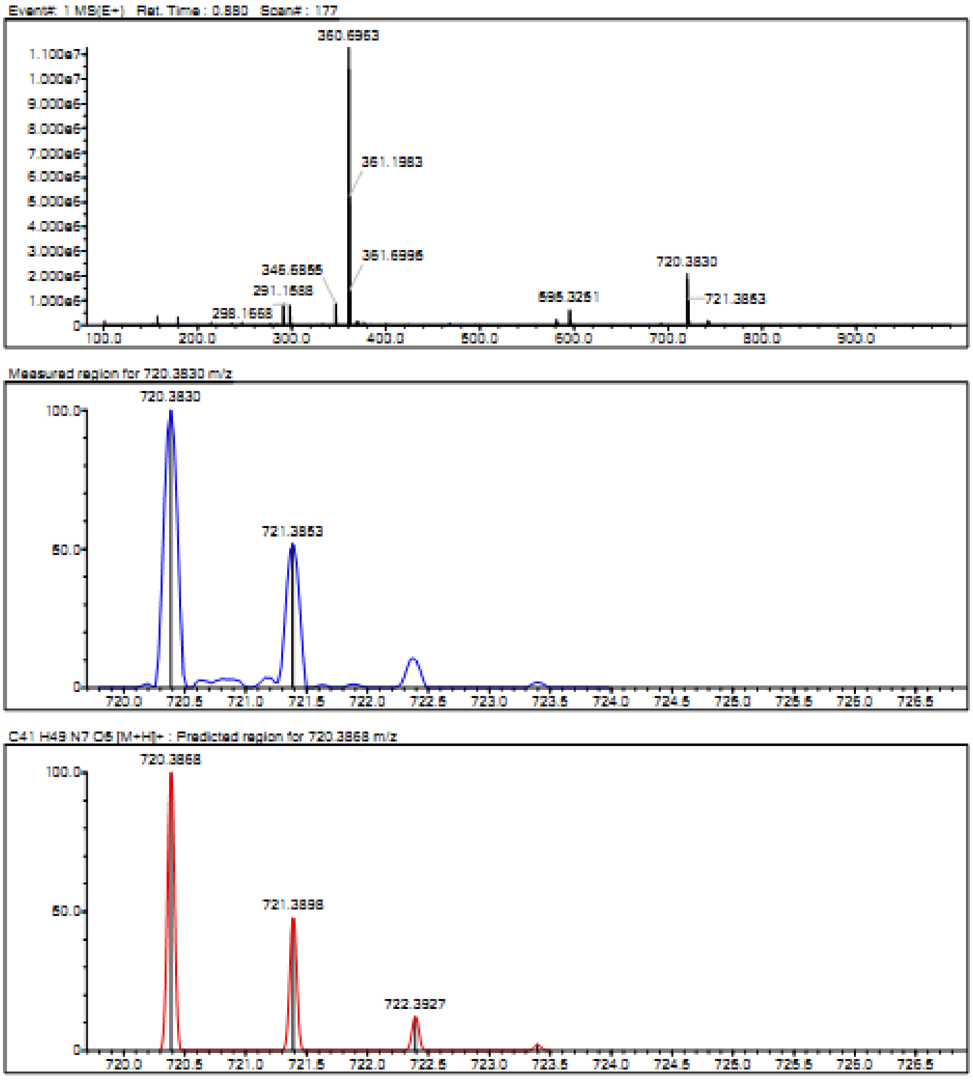

Rank	Score	Formula (M)	Ion	Meas. m/z	Pred. m/z	Df. (mDa)	Df. (ppm)	Iso	DBE
1	74.64	C41 H49 N7 O5	[M+H]+	720.3830	720.3868	-3.8	-5.27	85.60	21.0

Figure S27. HRMS spectrum of **14**

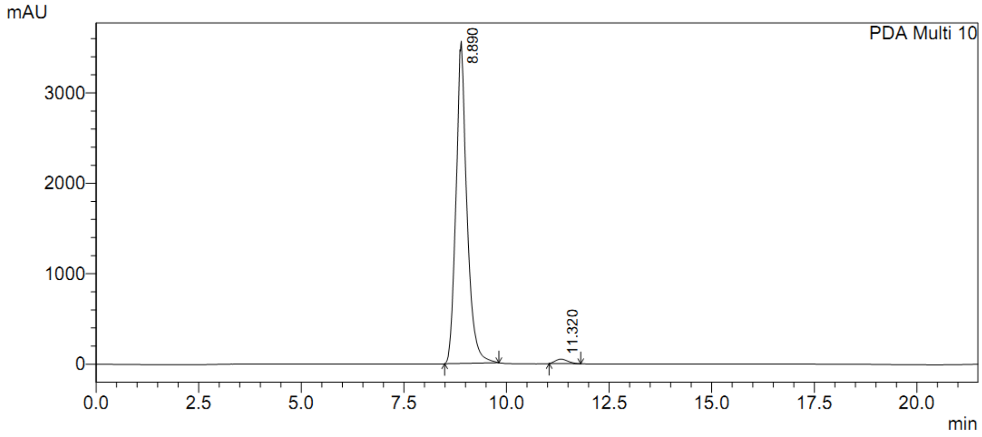

Figure S28. HPLC analysis of **14**

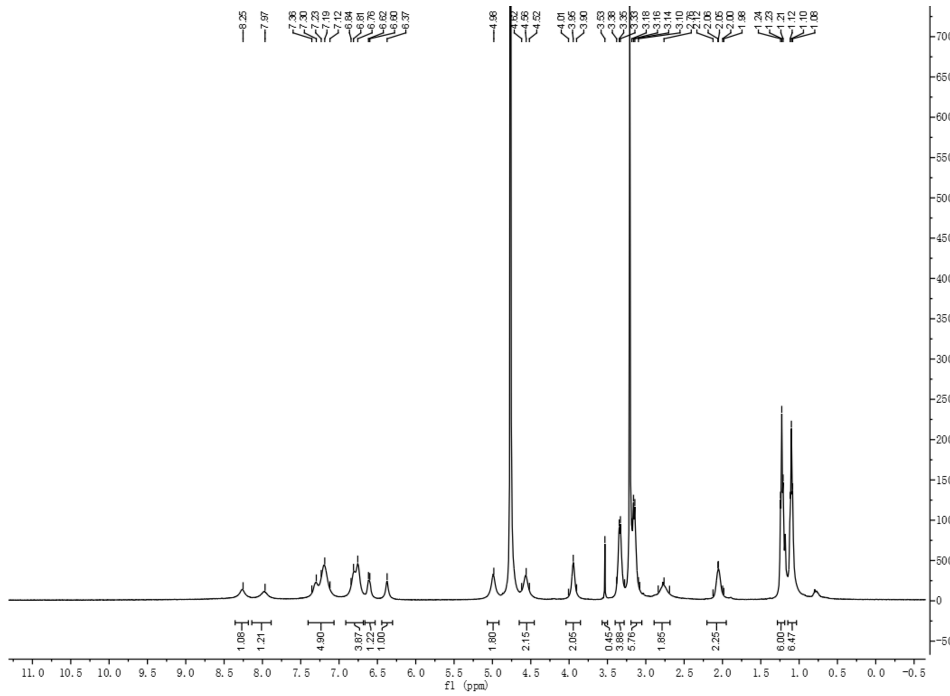

Figure S29. ^{1}H NMR spectrum of **15**

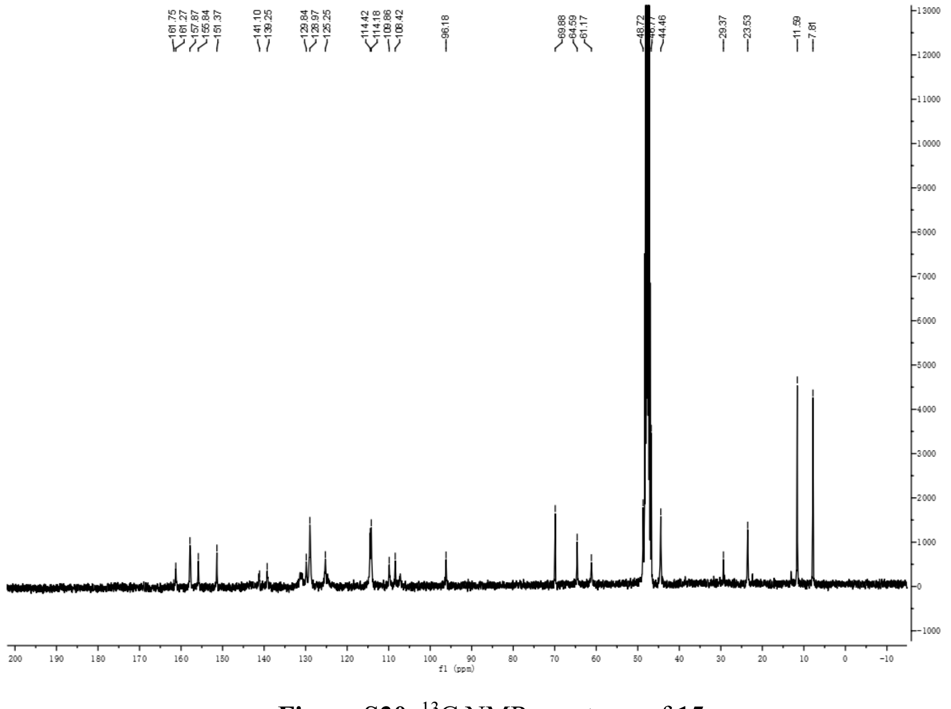

Figure S30. ^{13}C NMR spectrum of **15**

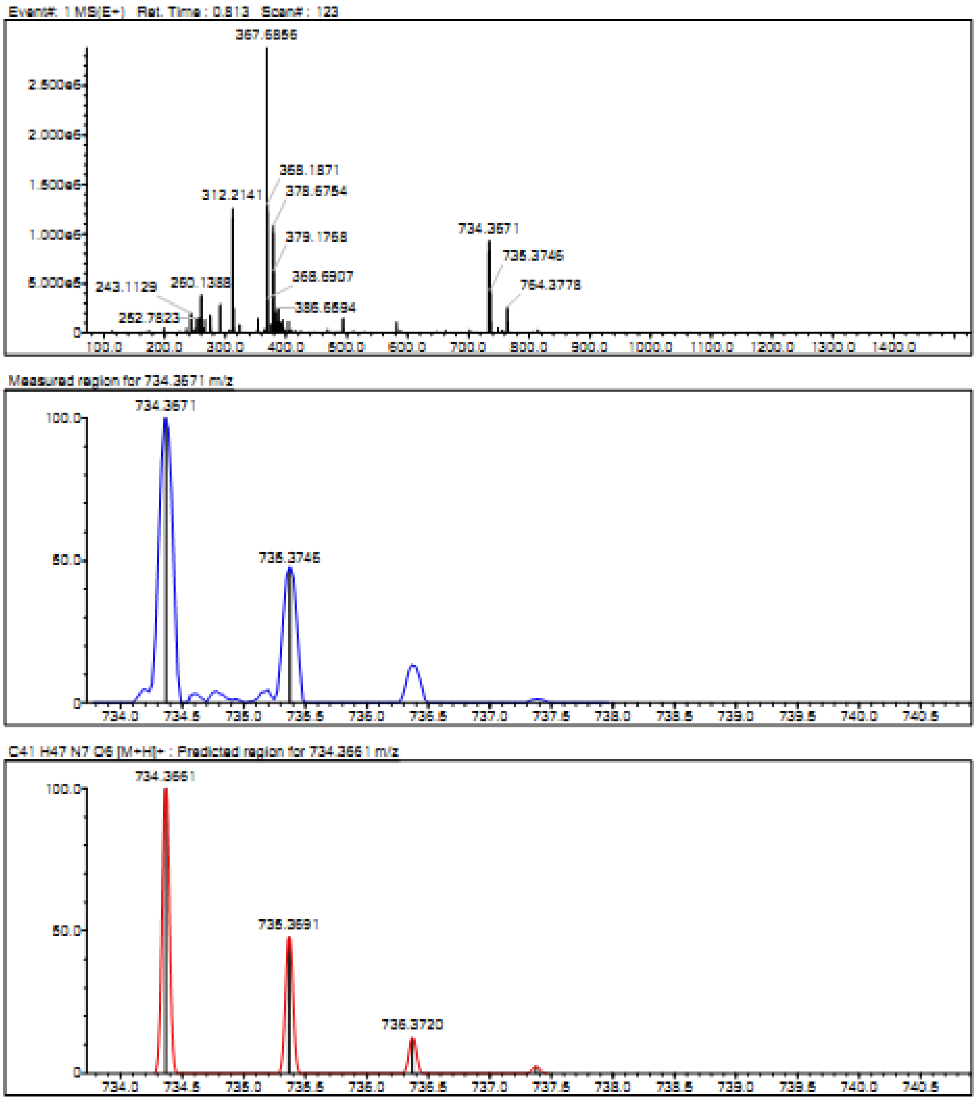

Rank	Score	Formula (M)	Ion	Meas. m/z	Pred. m/z	Df. (mDa)	Df. (ppm)	Iso	DBE
5	95.45	C41 H47 N7 O6	[M+H]+	734.3571	734.3561	1.0	1.36	96.33	22.0

Figure S31. HRMS spectrum of **15**

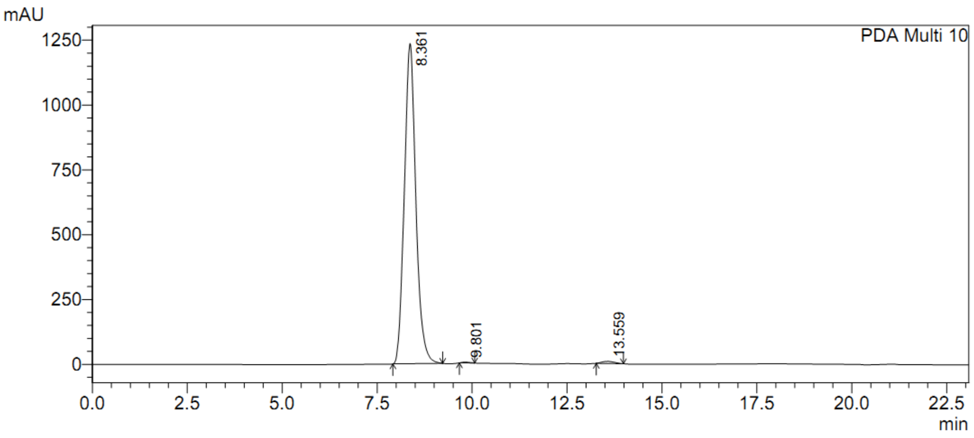

Figure S32. HPLC analysis of **15**

2. Other Supporting Table, Spectra and Graphs

Table S1. DNA samples used in the present study.

Name	Sequence (from 5' to 3')	Structure in K^+ Solution
KRAS	AGGGCGGTGTGGGAAGAGGGAAGAGGGGGAGG	Parallel G4
Pu22	TGAGGGTGGGTAGGGTGGGTAA	Parallel G4
bcl-2	GGGCGGGCGCGGGAGGAAGGGGGCGGG	Parallel G4
c-kit2	GGGCGGGCGCGAGGGAGGGG	Parallel G4
HT-L2H	TTGGGTTAGGGTTAGGGTTAGGGA	Hybrid-Type G4
htg22	AGGGTTAGGGTTAGGGTTAGGG	Hybrid-Type G4
HRAS	TCGGGTTGCGGGCGCAGGGCACGGGCG	Antiparallel G4
TBA	GGTTGGTGTGGTTGG	Antiparallel G4
c-kit3	GGCGAGGAGGGGCGTGGCCGGC	Antiparallel G4
TAA	$T21/(A21)_2$	Triplex DNA
ds26	CAATCGGATCGAATTCGATCCGATTG	Double-Stranded DNA
ctDNA	Calf Thymus DNA	Double-Stranded DNA
Py22	TTACCCACCCTACCCACCCTCA	Single-Stranded DNA
A21	AAAAAAAAAAAAAAAAAAAAA	Single-Stranded DNA
T21	TTTTTTTTTTTTTTTTTTTTT	Single-Stranded DNA
SPR-HRAS	biotin-AGGGTTAGGGTTAGGGTTAGGG	Antiparallel G4
SPR-c-kit2	biotin-GGGCGGGCGCGAGGGAGGGG	Parallel G4
c-kit2-Ap4	GGGApGGGCGCGAGGGAGGGG	Parallel G4
c-kit2-Ap12	GGGCGGGCGCGApGGGAGGGG	Parallel G4
c-kit2-Ap16	GGGCGGGCGCGAGGGApGGGG	Parallel G4
HRAS-Ap7	TCGGGTApGCGGGCGCAGGGCACGGGCG	Antiparallel G4
HRAS-Ap16	TCGGGTTGCGGGCGCApGGGCACGGGCG	Antiparallel G4
HRAS-Ap21	TCGGGTTGCGGGCGCAGGGCApCGGGCG	Antiparallel G4

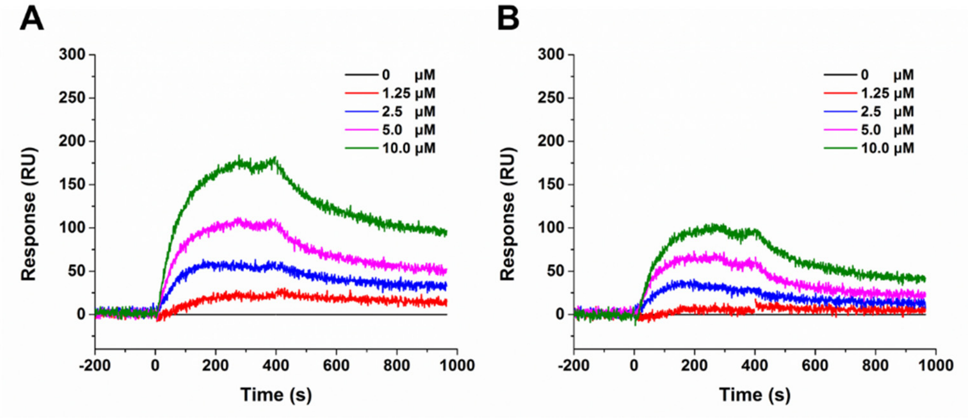

Figure S33. SPR sensorgrams of alkyne **1** with different oligonucleotides: (A) parallel G-quadruplex c-kit2, (B) antiparallel G-quadruplex HRAS.

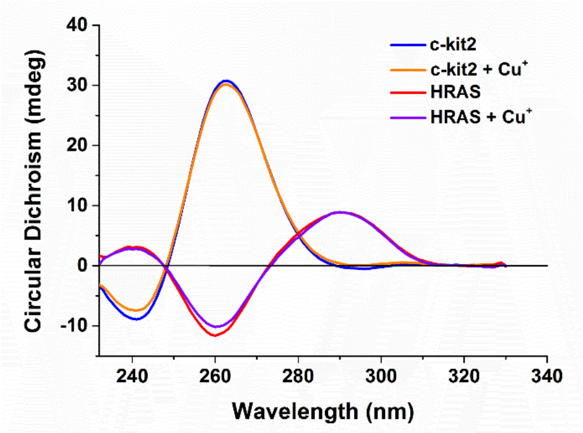

Figure S34. CD spectra of 12.5 µM G-quadruplex-forming oligonucleotides c-kit2 and HRAS in 10 mM Tris-HCl buffer, 100 mM KCl, pH 7.2, with and without 50 µM Cu^{I}.

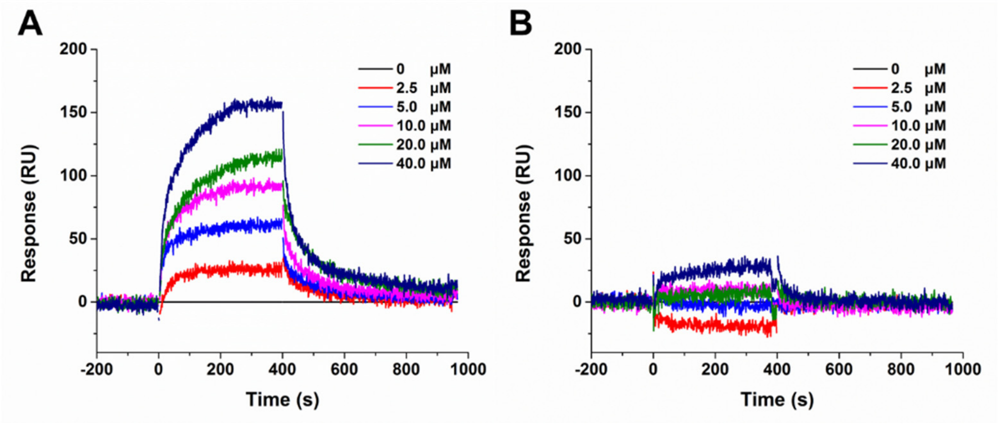

Figure S35. SPR sensorgrams of compound **15** with different oligonucleotides: (A) parallel G-quadruplex c-kit2, (B) antiparallel G-quadruplex HRAS.

Table S2. The fluorescence quantum yields of **15** with different nucleic acids [a]

Sample	Φ_F	Relative Φ_F
Tris	0.009	1
KRAS	0.523	58
pu22	0.442	49
c-kit2	0.440	49
bcl-2	0.419	47
HT-2LH	0.073	8
HRAS	0.059	7
htg22	0.042	5
TBA	0.032	4
c-kit3	0.060	7
TAA	0.047	5
ds26	0.027	3
ctDNA	0.017	2
Py22	0.027	3
A21	0.031	3
T21	0.032	4

[a] 1 µM of **15** and 10 µM of each sample were used in the determination of Φ_F.

Table S3. Detection limits of **15**, **IZCM-1** and **IZCM-7** for different G-quadruplexes in solution

Nucleic Acid	LOD (nM)		
	**15**	**IZCM-1**	**IZCM-7**
KRAS	8	13	3
Pu22	9	17	8
c-kit2	10	25	9

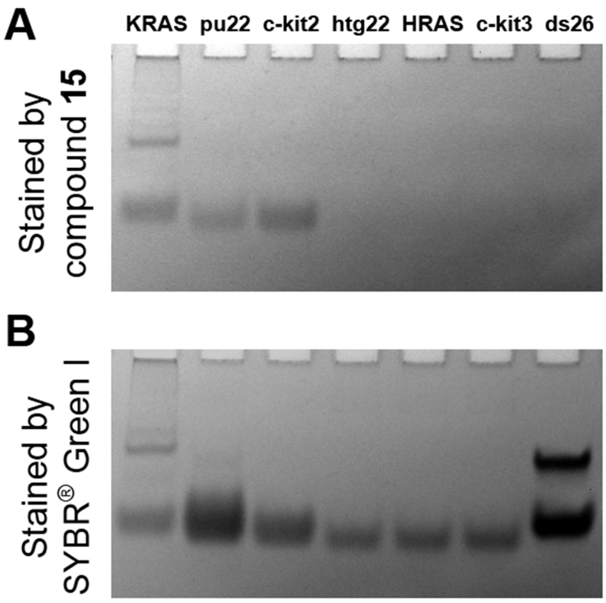

Figure S36. Staining of G-quadruplexes (KRAS, pu22, c-kit2, htg22, HRAS and c-kit3) and double-stranded DNA ds26 by (A) compound **15** and (B) SYBR® Green I. Conditions: 10 μL of nucleic acids were loaded onto 20% acrylamide in 1 × TBE buffer containing 100 mM KCl. The concentrations of all the nucleic acids were 5 μM.

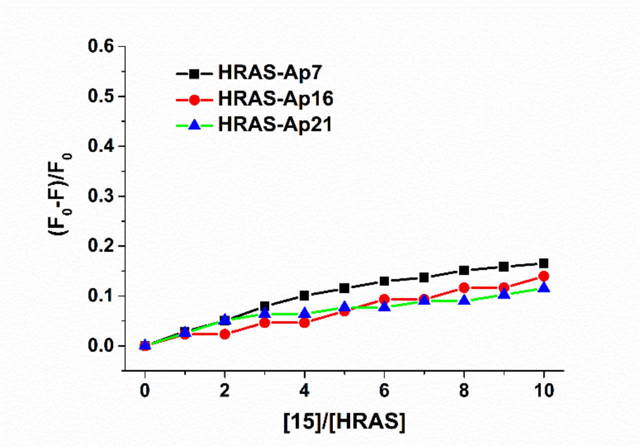

Figure S37. Plot of normalized fluorescence intensity at 375 nm of 1 μM 2-Ap individually labeled HRAS versus binding ratio of [**15**]/[c-kit2], λ_{ex} = 305 nm.

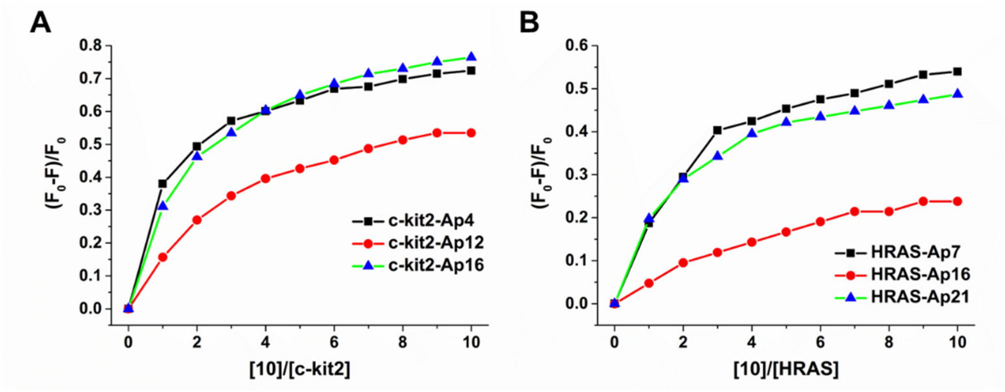

Figure S38. Fluorescence titrations of 2-AP labeled G-quadruplexes with stepwise addition of compound **10** in Tris-HCl buffer, 100 mM KCl, pH 7.2. (A) Plot of normalized fluorescence intensity at 375 nm of 1 μM 2-Ap individually labeled c-kit2 versus binding ratio of [**10**]/[c-kit2]. (B) Plot of normalized fluorescence intensity at 375 nm of 1 μM 2-Ap individually labeled htg22 versus binding ratio of [**10**]/[HRAS], λ_{ex} = 305 nm.
